# Supplementary material for: Synthesizing perspectives: Crafting an Interdisciplinary view of social media’s impact on young people’s mental health
Source: PLoS One. 2024 Jul 15;19(7):e0307164. doi: 10.1371/journal.pone.0307164 (PMC11249244; doi:10.1371/journal.pone.0307164)
Supplement: S1 Table — (DOCX) [file pone.0307164.s001.docx]

**S1 Table. Instructions for Multidisciplinary Analysis Using ChatGPT-4 (Used separately for each discipline.**

| **Analysis Stage** | **Specific Instructions** |
| --- | --- |
| **1. Dataset Introduction and Themes Identification:** | **Introduction:** Begin with a concise overview of the dataset, detailing its origin, nature, and any relevant contextual information. Specify the disciplinary and sub-disciplinary focus to guide the thematic analysis. |
|  | **Themes Identification:** Using the specified discipline as defined by its sub-discipline, analyse the dataset to uncover key themes. This process involves Braun and Clarke's five steps:   - **Familiarising yourself with your data:** Immerse yourself in the dataset, noting initial ideas and patterns. - **Generating initial codes:** Systematically code the data for significant features, keeping the disciplinary focus in mind. - **Searching for themes:** Organise codes into potential themes that capture something important about the data in relation to the research question and disciplinary focus. - **Reviewing themes:** Check the themes against the dataset, ensuring they form a coherent pattern and accurately represent the data. - **Defining and naming themes:** Refine each theme's specifics, clearly defining and naming them to reflect their essence. |
| **2. Quotes Extraction (follow-up instruction):** | Request two illustrative quotes per identified theme. Ask for an explanation of why each quote is representative, demanding a justification that underscores how the quote exemplifies the associated theme. |
