# Supplementary material for: Synthesizing perspectives: Crafting an Interdisciplinary view of social media’s impact on young people’s mental health
Source: PLoS One. 2024 Jul 15;19(7):e0307164. doi: 10.1371/journal.pone.0307164 (PMC11249244; doi:10.1371/journal.pone.0307164)
Supplement: S2 Table — (DOCX) [file pone.0307164.s002.docx]

**S2 Table. Disciplinary and Sub-disciplinary Frameworks for Thematic Analysis with a rationale for inclusion.**

| Discipline | Subdisciplines |
| --- | --- |
| Sociology | **Social Theory**: Offers frameworks for understanding how social media shapes young people's interactions and perceptions, impacting their mental health. **Social Research Methods**: Provides robust methodologies for analysing the influence of social media on the mental health of young individuals. **Cultural Sociology**: Explores the impact of social media-driven cultural norms and values on the mental health of children and adolescents. **Political Sociology**: Investigates the influence of social media on young people’s political awareness and its subsequent effects on their mental well-being. **Sociology of Religion**: Examines the interplay between social media, religious beliefs, and their collective impact on the mental health of young individuals. **Economic Sociology**: Studies how economic behaviours influenced by social media affect young people's mental health. **Urban Sociology**: Analyses the unique mental health challenges of urban young people in the context of their social media usage. **Rural Sociology**: Focuses on the mental health effects of social media among young individuals in rural settings, where social dynamics can be different. **Sociology of Education**: Looks at how social media influences educational environments and impacts student mental health. **Sociology of Health and Illness**: Offers insights into how social factors related to social media use affect the mental health of young people. **Industrial Sociology and Work**: Investigates how social media shapes young people’s perceptions of work and their mental health in the context of future career aspirations. **Sociology of Gender and Sexuality**: Analyses how social media influences young people's understanding of gender and sexuality, impacting their mental health. |
| Psychology | **Clinical Psychology**: Crucial for understanding and addressing potential mental disorders in young people exacerbated by social media use. **Cognitive Psychology**: Helps in exploring how social media affects cognitive processes like attention, memory, and problem-solving in young people. **Developmental Psychology**: Offers insights into how social media impacts the psychological development of children and adolescents. **Forensic Psychology**: Provides perspectives on legal and ethical issues related to social media use and its mental health implications in young people. **Health Psychology**: Investigates the interplay between social media use and physical and mental health in young individuals. **Industrial-Organisational Psychology**: Examines the impact of social media on young people's behaviours and attitudes in organisational settings. **Neuropsychology**: Studies the relationship between brain function and the impact of social media use on young people's behaviour. **Social Psychology**: Essential for understanding how social media influences individual behaviour and interpersonal relationships among young people. **Sports Psychology**: Explores the influence of social media on young athletes' mental health and performance. |
| **Education Studies** | **Curriculum and Instruction**: Important for integrating knowledge about social media's impact on mental health into educational programs. **Educational Psychology**: Provides insights into how social media influences learning and mental well-being in educational settings. **Special Education**: Addresses how social media impacts students with disabilities and their mental health. **Educational Leadership and Administration**: Essential for developing policies and strategies in educational settings regarding social media use and its impact on student mental health. **Educational Policy and Reform**: Focuses on shaping educational policies that consider the mental health implications of social media use among students. **Sociology of Education**: Offers insights into the societal and educational aspects of social media use and its impact on student mental health. |
| **Political Science** | **Comparative Politics**: Analyses how different political systems respond to the challenges of social media use among young people and its impact on mental health. **International Relations**: Looks at the global dynamics of social media and its influence on the mental health of young people across different cultures. **Political Theory**: Provides a theoretical framework for understanding the ethical and political implications of social media use on young people's mental health. **Public Administration and Policy**: Essential for devising policies that address the mental health impact of social media use among young people. **Political Economy**: Examines the interplay between economic policies and social media's influence on the mental health of young people. |
| **Philosophy** | **Metaphysics**: Offers a framework for understanding the existential questions raised by social media use and its impact on the mental health of young people. **Epistemology**: Explores how knowledge and beliefs formed through social media influence the mental health of young individuals. **Ethics**: Critical for evaluating the moral implications of social media use on the mental health of children and adolescents. **Logic**: Helps in constructing coherent arguments about the impact of social media on the mental health of young people. **Aesthetics**: Studies the influence of social media aesthetics on young people’s perceptions and mental health. **Political Philosophy**: Analyses social media's impact on young people's mental health from a perspective of justice, rights, and governance. |
| **Media Studies** | **Media Theory and Analysis**: Investigates how media content and representations on social media platforms influence the mental health of young people. **Media Production**: Looks at how the creation and dissemination of media content on social media platforms impact young people's mental health. **Media History**: Provides context for understanding the evolution of media and its impact on the mental health of young people. **Media Technology and Digital Media**: Studies the influence of social media technologies on the mental health of young individuals. **Media Law and Policy**: Examines the legal and ethical considerations of social media use and its impact on the mental health of young people. **Audience Studies**: Focuses on how young audiences interact with and are affected by social media, impacting their mental health. **Media and Cultural Studies**: Explores the role of media in shaping young people’s cultural and social identities and its impact on mental health. **Global and Transnational Media**: Looks at the global impact of social media on the mental health of young people across different cultures. |
| **Linguistics** | **Phonetics and Phonology**: Studies how the sounds and speech patterns in social media communication affect young people's mental health. **Morphology**: Examines how the structure of language in social media content influences young people's mental health. **Syntax**: Analyses how sentence structure in social media communication impacts the mental health of young users. **Semantics**: Investigates the impact of meaning and interpretation of social media content on young people’s mental health. **Pragmatics**: Looks at how the use and context of language in social media affects the mental health of young people. **Sociolinguistics**: Explores the relationship between language use on social media and its impact on the mental health of young people. **Psycholinguistics**: Studies the psychological aspects of language use on social media and its impact on young people’s mental health. |
| **Social Work** | **Clinical Social Work**: Focuses on identifying and treating mental health issues in young people influenced by social media use. **Community Practice**: Involves addressing the broader social implications of social media on young people’s mental health in community settings. **Social Work Administration**: Looks at managing social services that address the mental health impacts of social media on young people. **Policy Practice**: Involves developing social policies that address the mental health consequences of social media use among young people. **Child, Family, and School Social Work**: Focuses on the impact of social media on the mental health of children and adolescents in family and school contexts. |
| **Anthropology** | **Cultural Anthropology**: Studies how social media as a cultural phenomenon influences the mental health of young people. **Biological Anthropology**: Examines the biological and evolutionary factors affecting young people's mental health in the context of social media use. **Archaeology**: Provides a historical perspective on how past human social interactions compare to modern social media dynamics affecting mental health. **Linguistic Anthropology**: Investigates how language use on social media platforms influences the mental health of young people. **Social Anthropology**: Analyses the social structures and institutions influenced by social media and their impact on young people’s mental health. |
| **Health Sciences** | **Biomedical Sciences**: Examines the biological and physiological aspects of how social media use affects the mental health of young people. **Clinical Medicine**: Focuses on diagnosing and treating health issues related to social media use in young people. **Nursing and Allied Health**: Looks at the role of healthcare professionals in addressing the impacts of social media on young people’s mental health. **Public Health**: Studies the broader public health implications of social media use on the mental health of young populations. **Nutrition and Dietetics**: Examines the correlation between social media use, dietary habits, and mental health in young individuals. |
