# Supplementary material for: Synthesizing perspectives: Crafting an Interdisciplinary view of social media’s impact on young people’s mental health
Source: PLoS One. 2024 Jul 15;19(7):e0307164. doi: 10.1371/journal.pone.0307164 (PMC11249244; doi:10.1371/journal.pone.0307164)
Supplement: S3 Table — (DOCX) [file pone.0307164.s003.docx]

**S3 Table. Instructions for Multidisciplinary Analysis Using ChatGPT-4 (Separately for primary and secondary schools).**

| **Stage of Analysis** | **Instructions given to ChatGPT-4** |
| --- | --- |
| **1. Review of Multidisciplinary Analyses and Identification of Superordinate Themes** | Start by systematically reviewing the thematic analyses conducted across 10 academic disciplines. Understand the key themes identified within each discipline.  Analyse these themes to identify overarching, superordinate themes that capture the essence of findings across disciplines, using Braun and Clarke's methodological steps. |
| **2. Mapping Themes to Super-Ordinate Themes** | For each superordinate theme identified, specify which themes from the individual disciplinary analyses contribute to it. Detail how these themes align or contrast within each superordinate theme, providing a nuanced understanding of the dataset's multifaceted nature. |
| **3. Comprehensive Analysis of Super-Ordinate Themes** | Conduct an in-depth analysis of each superordinate theme, integrating diverse academic insights from the individual analyses. This exploration should offer a multidimensional perspective on the dataset, enriching the understanding of the researched phenomenon. |
| **4. Development of an Overarching Theory for Each Super-Ordinate Theme** | Based on the comprehensive analysis, develop an overarching theory for each superordinate theme. This theory should synthesise the different academic perspectives, reflecting the complexity and multidisciplinary nature of the findings. |
