# Supplementary material for: Synthesizing perspectives: Crafting an Interdisciplinary view of social media’s impact on young people’s mental health
Source: PLoS One. 2024 Jul 15;19(7):e0307164. doi: 10.1371/journal.pone.0307164 (PMC11249244; doi:10.1371/journal.pone.0307164)
Supplement: S4 Table — (DOCX) [file pone.0307164.s004.docx]

**S4 Table. Thematic Analysis of Primary School Headteacher Narratives around Social Media effects on young people’s mental health.**

| Academic Domain | Theme | Quote |  | Rationale |
| --- | --- | --- | --- | --- |
| Psychological | **Influence on Self-Perception and Identity Formation**: Social media significantly shapes how young children perceive themselves and their place in the world, which is a crucial aspect of developmental psychology. | "I think Social Media sites such as [*named social media*] have an enormous influence. Partly because of the unrealistic expectations of what a 'good life' looks like (always travelling having expensive luxury goods etc.) and partly because of a perceived 'standard' of what people should look like - this is especially prevalent amongst young girls." |  | Emphasises social media's influence on young peoples' self-concept and identity development, shaping self-perception and self-esteem through idealised life and beauty portrayals, potentially causing psychological distress. |
|  |  | "Self-esteem and body image are adversely affected by the unrealistic expectations portrayed on social media. This phenomenon is observed equally in boys and girls. There's also a significant issue with FOMO (Fear of Missing Out) where young individuals are using social media late into the night because they worry about missing out on events or updates despite our efforts to educate them on safe usage and our frequent communication with parents." |  | Discusses social media's role in shaping self-esteem and body image, key elements in self-perception, and highlights its contribution to distorted self-images and FOMO, crucial in developmental psychology. |
|  | **Exposure to Inappropriate Content and Cyberbullying**: The accessibility of harmful content and the prevalence of cyberbullying on social media platforms are major concerns, resonating with the principles of clinical and health psychology. | "Watching content which is violent and filled with aggressive language can affect how a young child views the world. They might copy this behaviour particularly if it is presented by a popular influencer... " |  | Influence of inappropriate content exposure on children's psychological development, particularly the role of violent content and influencer amplification, aligning with developmental and clinical psychology principles. |
|  |  | "In cases of cyberbullying a child may never get a break from harassment as bullies can target them even late at night... There are also questionable websites that encourage children to engage in inappropriate or even illegal activities, potentially causing significant harm to a child's mental health. Unfortunately, these negative aspects of social media don't disappear, and the constant exposure can be detrimental” |  | Highlights the enduring and invasive cyberbullying phenomenon, especially relevant in child and adolescent psychology. It emphasises ongoing stress, mental health concerns, low self-esteem, and inadequacy, central to clinical and health psychology in young people. |
|  | **Social Skills and Peer Interaction**: The impact of social media on children's real-world social skills and interactions is a key theme, related to social and developmental psychology. | "I work with young children aged 5-7 and they are much more aware of things that aren't age appropriate eg violence, swearing, sexualised images. This often comes out in their play and interactions with peers which causes difficulty in building healthy relationships and age-appropriate play. Children have also expressed upset about things their parents have shared on social media that features them - e.g. pictures where they look a bit silly or are having a tantrum." |  | This quote underscores the psychological impact of inappropriate social media content on children's development, particularly its effects on play, peer interactions, and self-esteem. |
|  |  | "SM (Social media) asserts peer pressure on young people and young adults by implying that there is a standard of perfection which they should be aspiring to in terms of body image and lifestyle." |  | This quote aligns with psychological concerns regarding social media's influence on young peoples’ self-image and peer dynamics. It highlights the pressure to conform, impacting self-esteem and social comparison, crucial in social and developmental psychology. |
| Sociological | **Social Comparison and Self-Perception**: This theme reflects the idea that social media influences young people's self-image and self-worth by exposing them to unrealistic standards and expectations. | “Through social media, they compare themselves to the unrealistic and often untrue lives of other people on social media which causes them to feel bad about their own lives.” |  | Highlighting how social media shapes children's self-perception and comparisons. It aligns with media influence theories, reflecting the 'looking-glass self' concept. |
|  |  | "A perfect world is often portrayed through social media and children and young people can be drawn into thinking their life should be like that... Self-esteem can be damaged. Long hours spent staring at phones or tablets whilst interacting with social media also affects mental health and well-being as they spend less time socialising in person, more time indoors, less time communicating with others properly etc..." |  | Emphasising how social media fosters an idealised world, affecting children's self-esteem through social comparison. It also highlights shifts in communication and relationships, reflecting sociological tech and society concerns. |
|  | **Digital Peer Interaction and Cyberbullying**: Definition This theme involves the impact of social media on peer relationships, including the prevalence of cyberbullying and the pressure to maintain online social connections. | "I think Social Media sites such as [*named social media*] have an enormous influence. [...] I also think social sites where young people can chat to each other can have a big influence as it's much easier to say mean things behind a screen." |  | Discussing social media's influence on young people, focusing on peer dynamics and cyberbullying. It underscores anonymity's role in negative online peer interactions. |
|  |  | "An example we've been dealing with in school: use of [social media forum] - children can't get away from their peers at any time. Issues that have happened in school or in the community then carry on at home and well into the night. [...] Children think that they must be on social media in order to be part of a social group..." |  | Highlights the persistent digital peer interactions on social media, reflecting sociological group dynamics, social identity, and socio-cultural pressures related to cyberbullying. |
|  | **Constant Connectivity and Mental Overload**: Reflecting the notion that the always-on nature of social media creates an environment of constant connectivity, leading to mental overload and stress. | “It puts a lot of pressure on children both to be available to respond immediately and to be on there in the first place to not miss out.” |  | Highlights the sociological concern of constant connectivity's pressure on children, creating an environment of instant responsiveness and FOMO, reflecting mental overload and its associated stress and anxiety. |
|  |  | “I think that for many children it is modelled at home with parents and older sibling sharing content and using phones- games social media as a 'baby sitter' = keeps the children quiet.” |  | Delves into the sociological impact of social media and digital devices in family dynamics, emphasising their role in modelling and normalising constant connectivity, contributing to mental overload, stress, and cognitive fatigue. |
| Education Studies | **Digital Literacy and Critical Thinking**: This theme focuses on the ability of students to critically assess information found on social media and the importance of teaching these skills to young people. It underlines the need for educational strategies that enable students to discern reliable information online. | "More studies have shown that social media in general is not as a positive interaction as we first believed. With false information and clickbait stories being passed around all people are being misled. We encourage our children to question what they see and read and to talk about things that they discover and do not understand. Children like adults are easily misled by those who groom them into believing what they are selling and it is never for the better of the children. Social media should be banned for children younger than 15. They will have less stress about friends their bodies food and trends." |  | Underscores the educational concern of misinformation and clickbait on social media, emphasising the importance of teaching students critical assessment and digital literacy skills to discern reliable information. |
|  |  | "It plays a significant part! Our children spend a good deal of their spare time using a variety of platforms and generally tend to trust / believe what they see / are told. We do teach online safety at school however it is increasingly hard to compete with the power of social media." |  | Highlights the impact of social media on children's trust in online content, emphasising the necessity of teaching online safety and critical thinking, integral to Digital Literacy education. It underscores the need for improved educational strategies to navigate online information. |
|  | **Social and Emotional Learning**: This theme involves the development of skills for healthy interaction and emotional regulation, influenced by social media use. It underscores the impact of online interactions on students' emotional well-being and social skills. | "Social Media sites such as [*named social media*] have an enormous influence. Partly because of the unrealistic expectations of what a 'good life' looks like... and partly because of a perceived 'standard' of what people should look like - this is especially prevalent amongst young girls." |  | Underscores how social media affects children's self-image and well-being, especially girls, emphasising the importance of SEL programs teaching healthy self-perception, emotional regulation, and resilience against unrealistic standards, aligning with educational studies. |
|  |  | "Children like adults are easily misled by those who groom them into believing what they are selling and it is never for the better of the children. Social media should be banned for children younger than 15. They will have less stress about friends their bodies food and trends." |  | Stresses children's vulnerability to misinformation and online grooming, underscoring the importance of Social and Emotional Learning programs that teach critical thinking, safety, stress management, and healthy relationships in the context of educational studies. |
|  | **Cyberbullying and Online Safety**: This theme highlights the prevalence of cyberbullying and the importance of creating a safe online environment for students. It emphasises the role of schools in educating about online safety and addressing cyberbullying. | "We have had incidents of self harming, school refusers, children talking online to unknown strangers, and depression in children due to what they have seen and experienced online." |  | Highlights the adverse effects of unchecked social media on children, encompassing self-harm, school avoidance, and depression. It underscores the urgency for robust education on online safety and cyberbullying in educational studies. |
|  |  | "Children think that they must be on social media in order to be part of a social group which means more children are having phones at an early age." |  | Highlights the societal pressures pushing children into premature social media use, increasing their susceptibility to cyberbullying and online risks. It underscores the necessity for educational interventions addressing these pressures and promoting responsible social media use in educational studies. |
|  | **Influence on Self-Perception and Identity**: This theme reflects the impact of social media on young people's self-image and identity formation. It demonstrates how social media can affect students' perceptions of themselves and others. | "The pressure on girls to look a certain way is profound and we see a significant drop in self esteem and confidence once the children reach certain ages; usually around 9 years old. Although it is normal for children to compare themselves to others and want to fit in this is magnified by the use of social media with influencers presenting abnormal standards for children to feel they need to live up to.” |  | Pertinent in Educational Studies as it addresses social media's impact on children's self-perception, especially girls. It highlights decreased self-esteem due to unrealistic standards, crucial for understanding identity and mental health. |
|  |  | “It gives them ideals to look up which are impossible to maintain all the time. It does not acknowledge the ups and downs of everyday life at times. Yet at other times it can provide too much personal information that may misrepresent or misinform creating further mental health problems”. |  | Significant in Educational Studies, discussing social media's influence on self-perception and identity, encompassing self-doubt, low self-esteem, and unrealistic standards. It underscores psychological implications and the internalisation of harmful standards. |
| Political Science | **Parental and Community Involvement**: This theme emphasises the importance of parental and community engagement in managing the impact of social media on young people. It suggests a collaborative approach involving parents, educators, and the wider community. | "We give quite a lot of input repeating - never press send if you wouldn't be comfortable for your grandparents to read what you have written. We also see the impact of over use of technology to babysit and that parents are on their phones so not engaging orally with their children. We send out a monthly online safety newsletter and encourage parents at any opportunity to delay giving smart phones (old style fine) - definitely setting boundaries charging them overnight in living spaces not bedrooms. Like most things - in moderation is best." |  | Underscores educational institutions' role in promoting responsible social media usage, with a focus on parental involvement. It aligns with societal responsibility and suggests the necessity of supportive policies in political science. |
|  |  | "I think we should look at parental use of social media and how it impacts on children as well as children's own use of social media. Every school has a toxic parent [*named social media*] group or two and this negatively affects the children. When children are aware that parents don't value or respect a teacher the child can disengage with learning - and that in turn affects confidence, self-esteem." |  | Highlights parental influence on children's social media behaviours, emphasising community norms. In political science, it underscores the need for community-wide education addressing both young people and adult online behaviour. |
|  | **Policy Impact on Social Behaviour**: This theme explores how social media, as a modern phenomenon, impacts societal norms and individual behaviour. It aligns with Public Administration and Policy in political science, focusing on the consequences of widespread social media use on young peoples’ behaviour and mental health. | "More studies have shown that social media in general is not as a positive interaction as we first believed. With false information and clickbait stories being passed around all people are being misled. We encourage our children to question what they see and read and to talk about things that they discover and do not understand. Children like adults are easily misled by those who groom them into believing what they are selling and it is never for the better of the children. Social media should be banned for children younger than 15. They will have less stress about friends their bodies food and trends." |  | Highlights parental influence on children's social media behaviours, emphasising community norms. In political science, it underscores the need for community-wide education addressing both young people and adult online behaviour. |
|  |  | "Self-esteem and body image are adversely affected by the unrealistic expectations portrayed on social media. This phenomenon is observed equally in boys and girls. There's also a significant issue with FOMO (Fear of Missing Out) where young individuals are using social media late into the night because they worry about missing out on events or updates despite our efforts to educate them on safe usage and our frequent communication with parents. Additionally we've seen a substantial increase in bullying especially since the onset of the COVID-19 pandemic. Much of this bullying occurs on social media during the evening and late at night. Regrettably we find ourselves addressing these issues during the day as parents often don't want to take responsibility for them during the nighttime hours." |  | Emphasising the repercussions of social media on young peoples’ mental health and behaviours. It underscores the need for policy interventions in education, mental health support, and digital literacy to counteract negative social media effects, revealing the interplay of social media, societal norms, and policy. |
|  | **Globalised Cultural Influence**: This theme relates to International Relations and examines the global reach of social media platforms, influencing cultural norms and behaviour patterns across different countries, affecting young people's mental health and societal views. | "I think Social Media sites such as [*named social media*] have an enormous influence. Partly because of the unrealistic expectations of what a 'good life' looks like (always travelling, having expensive luxury goods, etc.) and partly because of a perceived 'standard' of what people should look like - this is especially prevalent amongst young girls." |  | Emphasises the global influence of social media sites to transcend cultural boundaries to impact young peoples’ mental health and societal perspectives, aligning with Internationalisation in Political Science. |
|  |  | "Social Media has a lot of influence on children, young people, and young adults alike. In children and young people especially, it can and has had an impact on their mental health in an extremely negative way, and I have seen this first hand. Working in a Primary School where children as young as seven have been able to access social media has been quite frightening." |  | Highlights social media's pervasive global influence on children of all ages and its impact on mental health. It underscores how international media platforms shape societal views and individual well-being, emphasising the universal accessibility of social media to young children across cultures, a concern that transcends national boundaries. |
|  | **Governance and Regulation of Digital Spaces**: Pertaining to Public Administration and Policy, this theme looks at the role of government and regulatory bodies in managing and overseeing digital spaces like social media, with a focus on protecting young users’ mental health. | "More studies have shown that social media in general is not as a positive interaction as we first believed. With false information and clickbait stories being passed around all people are being misled. [...] Social media should be banned for children younger than 15. They will have less stress about friends their bodies food and trends." |  | Underscores the challenges of misinformation and social media's adverse effects on children's mental health, implying the need for regulatory measures, such as age restrictions. It aligns with political science discussions on government and regulatory bodies' roles in safeguarding young digital users. |
|  |  | "Self-esteem and body image are adversely affected by the unrealistic expectations portrayed on social media. [...] Additionally we've seen a substantial increase in bullying especially since the onset of the COVID-19 pandemic. Much of this bullying occurs on social media during the evening and late at night. Regrettably we find ourselves addressing these issues during the day as parents often don't want to take responsibility for them during the nighttime hours." |  | Discusses social media's impact on self-esteem and cyberbullying, advocating for increased governance and regulation in digital spaces. It aligns with political science's call for government intervention to protect vulnerable populations like children. |
| Philosophy | **Perception and Reality:** This theme explores how social media shapes young people's understanding of reality and their perception of self and others. Social media often presents an unrealistic portrayal of life, affecting children's self-image and understanding of the world. | "It affects their perception of reality in terms of what is normal or not. It affects the amount and quality of their sleep. It is a negative effect generally." |  | Highlights how social media affects children's perception of reality, echoing philosophical discussions on digital environments' impact on our worldview and physical experiences. |
|  |  | "I think Social Media sites such as [*named social media*] have an enormous influence. Partly because of the unrealistic expectations of what a 'good life' looks like (always travelling, having expensive luxury goods etc.) and partly because of a perceived 'standard' of what people should look like - this is especially prevalent amongst young girls." |  | Examines social media's influence on unrealistic expectations, notably for young girls, tying into philosophical debates on external influences shaping self-perception, reality, and desires in the digital age. |
|  | **Behaviour and Morality**: This theme addresses the influence of social media on young people's moral values and behaviours. It includes concerns about cyberbullying, peer pressure, and the impact of social media on kindness and empathy. | Sometimes it feels in schools we are dealing with fall out from social media outside of school. Parents who themselves are prey to social media do not seem to recognise the harm it does to their children unless properly supervised. I think the internet and social communication has been a game changer and has opened up so many opportunities to our children but we must also be very mindful of the destructive nature it can have. Responses on social media often show a lack of regulation by children quick to respond in aggressive tones causing extreme hurt. This lack of regulation impacts themselves and others. “ |  | Delves into philosophical inquiries regarding behaviour, morality, and digital environments, focusing on social media's influence on young children's moral values, self-regulation, aggression, empathy, and kindness development. |
|  |  | "I think overall it has a detrimental effect on children when content is not monitored and time limited by adults. I have been teaching since 1993 and in recent years seen an increase in bullying, negative thoughts, inappropriate behaviours and although I cannot say definitively it is due to social media it does have an impact. There are worrying perceptions about appearance and individuals worrying about things they should not be thinking about. Childhood seems to be reducing with many children 'addicted' to their phones. I think that for many children it is modelled at home with parents and older siblings sharing content and using phones- games, social media as a 'baby sitter' = keeps the children quiet” |  | Highlights unsupervised social media's impact on children's behaviour and moral development, raising philosophical concerns about technology's ethics, addiction, childhood essence, moral education, and character formation. |
|  | **Cognitive and Emotional Development**: This theme delves into the impact of social media on cognitive skills and emotional development, including issues like attention span, critical thinking, and emotional regulation | “From a very young age children are beginning to high expectations in regard to things they should be doing wearing and look like. They cannot seem to distinguish between what’s on social media and reality. They are also using social media to help regulate their emotions which causes the brain to be constantly stimulated and therefore they are not giving their brains a break like playing outside. When children are asked to take a break from their devices more often than not they display distressing behaviour" |  | Underscores social media's influence on children's perception of reality, emotional regulation, and self-concept, touching on philosophical discussions about reality, perception, and cognitive-emotional development. |
|  |  | "Social media... means they don't get a break if there's been a falling out at school this is continued later in [*named social media*]. They often aren't mature enough to read the emotion behind messages so something that would be a little tease in person they find upsetting over social media. It makes them more susceptible to peer pressure. They often bring issues into school the next day which shows they've been worrying all night and still concerned the next day which can't be beneficial to their mental health.” |  | Explores the shift of social interactions to digital realms, prompting philosophical inquiries into digital identity, social cognition, emotional well-being, and resilience in a digital-centric society. |
|  | **Social Dynamics and Inclusion**: This theme examines the role of social media in shaping social structures, inclusion, and exclusion among young people. It covers how social media affects social interactions, friendships, and the sense of belonging. | " Social media has a significantly detrimental effect on children's mental health. We see constant pressures from social media in terms of how children feel they need to look and act. In addition, bullying and peer pressure via social media is a constant battle and we are always having to deal with issues between children in school based on something that has happened on social media. The pressure on girls to look a certain way is profound and we see a significant drop in self esteem and confidence once the children reach certain ages; usually around 9 years old. Although it is normal for children to compare themselves to others and want to fit in, this is magnified by the use of social media with influencers presenting abnormal standards for children to feel they need to live up to" |  | Delves into philosophical inquiries about digital identity, belonging, and existential aspects. It underscores social media's role in shaping children's self-perception, behaviour, and sense of belonging while raising concerns about exclusion and conformity. |
|  |  | "I think social media can have a detrimental influence on Young People's mental Health. An example we've been dealing with in school: use of [*named social media*] - children can't get away from their peers at any time. Issues that have happened in school or in the community then carry on at home and well into the night. Children in control of [named social media] groups will then do things like chuck their peers out of groups etc. This also happens within online gaming communities too. Children think that they must be on social media in order to be part of a social group which means more children are having phones at an early age.” |  | Examines the relentless nature of social media interactions, sparking philosophical inquiries into modern social relationships and community. It pertains to Social Dynamics and Inclusion, emphasising digital impact on children's mental health and belonging. |
| Media Studies | **Influence of Media on Self-Perception and Identity Formation**: Discusses how social media shapes children's self-image and identity. | "In my opinion I believe social media plays a huge role in the influence of what the child thinks and feels about themselves. Body image and the social media sites which influence the negative thoughts and feelings" |  | Highlighting social media's influence on children's self-perception, emphasising unrealistic standards, blurred reality, and the search for validation, central to understanding media's impact on identity and self-image. |
|  |  | " I think it can have quite a significant influence and often to young people's detriment. These days social media I think can be seen as something of a virtual extension of the playground where image is hugely important and it can so easily be used as a way of bullying unfortunately.” |  | Equating social media to a virtual playground with a focus on image and social status, addressing issues of digital-age bullying and image impact. |
|  | **Impact of Digital Communication on Social Skills**: Addresses how social media alters traditional communication and social interaction skills. | “I work in a primary school and I would say from about age 7/8 our children have a social media presence and awareness. They’ll freely discuss [*named social media*] they’ve watched as half of them at least have phones of their own and they’ll set up little WhatsApp groups to chat back and forth with each other. It can be very clear how often they’re using social media and how quickly they get into it and it becomes important in their lives. They become a bit manic about keeping up with their groups and those who don’t have access to social media feel adrift and a little bit desperate that they’re being left out" |  | Addresses the shift from face-to-face interactions to digital communication among primary-aged children, impacting social skills and behaviour development through overuse and dependency on social media. |
|  |  | “The FOMO develops especially quickly in a young brain. I perceive a decrease in ability to concentrate, to parse information, and self-motivation” |  | Examining the media psychological concept of 'Fear of Missing Out' (FOMO) and its effects on young individuals, including decreased concentration and self-motivation attributed to constant digital connectivity. |
|  | **Media Literacy and Digital Savvy**: Emphasises the need for children to critically understand and engage with digital content. | " Balancing the positive and negative aspects requires a combination of digital literacy, responsible platform use, and open communication between parents, educators, and young individuals.” |  | Underscores the significance of digital literacy and responsible platform usage, stressing the role of parental and educational guidance in navigating digital environments. |
|  |  | " Our children spend a good deal of their spare time using a variety of platforms and generally tend to trust / believe what they see / are told. We do teach online safety at school, however it is increasingly hard to compete with the power of social media. |  | By addressing the difficulties young children face in evaluating the credibility of content on social media platforms. |
|  | **Online Community and Social Support**: Explores the role of social media in creating support networks and a sense of belonging for children. | “I believe that social media has a wide, and growing, impact upon the mental health of our children. A great deal of this is positive. We live in a rural community, and our children are widely dispersed. This inhibits their ability to meet face to face, and so they use social media for social contact, even at a young age. We have seen that this brings numerous benefits.” |  | Recognising social media's role in facilitating social contact, particularly in cases of geographical distance hindering in-person interactions. |
|  |  | “I am aware of children sending and receiving messages late into the night, when they should be sleeping, keen not to miss out and those who are less regulated by their parents seem to influence others. the content that children access is often a concern and brings language and attitudes into school that cause concern. Children enjoy the friendships extending outside of school but this tends to be game-based with children playing the same online games together. This has positives and negatives due to the competitive nature of the games.” |  | Underscoring how social media and online gaming extend friendships, while examining both positive aspects (relationship maintenance) and negatives (exposure to harmful content, peer pressure). It emphasises the significance of online communities and their intricate dynamics, crucial in studying digital social support and community building. |
| Linguistics | **Psychological and Emotional Impact of Media Consumption**: Focuses on the mental health implications of social media usage. | “It reduces focus and conditions them for over stimulus. It is impacting on their ability to self-regulate or co-regulate, due to how much it is used by parents to 'quieten' their much younger children when they are dysregulated. It is impacting upon self-image and forcing an unhealthy comparison with too many different people and too many unrealistic presentations of the lives of others” |  | Addresses media's influence on cognitive processes crucial for language acquisition and usage, and highlights how it relates to socio-linguistic identity formation in social contexts. |
|  |  | "We have children who have impacted sleep because of their obsessional behaviours linked to social media use. We have children in school who have body image issues including some who are controlling their food intake because of what they have seen on social media. We have children who have unrealistic expectations around possessions and wants vs needs.” |  | Addressing media's impact on language and self-perception. Influencers and media set 'ideal' standards, shaping children's self-concept and language use in self-expression, a key sociolinguistic concern. |
|  | **Influence on Self-Perception and Body Image**: This theme explores how social media shapes children's views on beauty and self-worth. Social media often presents idealised images that can lead to unrealistic standards and negative self-comparison. | " The pressure on girls to look a certain way is profound and we see a significant drop in self esteem and confidence once the children reach certain ages; usually around 9 years old. Although it is normal for children to compare themselves to others and want to fit in, this is magnified by the use of social media with influencers presenting abnormal standards for children to feel they need to live up to.” |  | Addressing media's impact on language and self-perception. Influencers and media set 'ideal' standards, shaping children's self-concept and language use in self-expression, a key sociolinguistic concern. |
|  |  | “Social media has a negative influence on the body image that girls have of themselves, this is vastly due to models sizing and celebrities etc using photoshopping to make the "perfect skin, waistline or lips. Several children have been on the receiving end of negative things said to them either by a stranger or someone they know, either via a social media platform or an online community linked to gaming.” |  | Highlights social media's impact on children's body image and self-esteem, focusing on linguistic elements in comments and idealised images. It underscores the importance of studying linguistic communication of beauty and self-worth in shaping self-perception and body image in young people. |
|  | **Cyberbullying and Peer Pressure**: Addresses the impact of online bullying and the pressure to conform to peer expectations. Social media can be a platform for bullying and creating pressure to adhere to peer norms. | “Previously bullying and peer pressure were situations which mainly occurred at school or when with friendship groups in social situations. Now, with high access to phones and social media, the pressure is more present than ever. Make up videos, fitness videos, videos from influencers who suggest certain types of behaviour are constant and children and young people's mental health is in crisis because home is no longer the sage space it was and dangerous people such as [name of influencer] and his ideas about women have a far greater reach than ever before.” |  | Underscores social media's role in establishing unrealistic standards, fueling cyberbullying and peer pressure, shaped by linguistic communication modes (e.g., posts, comments). It also highlights the transformation of bullying and peer pressure due to constant online connectivity, of interest in digital communication and linguistics research. |
|  |  | “The amount of real life conflict between children that is caused by things that have happened on social media is significant, and it can lead to some really nasty bullying that would not otherwise occur in such a virulent and aggressive way (without the “keyboard warrior” mentality). Also, predators use social media a lot to communicate with victims so that is another issue affecting mental (and sometimes physical) health in young people.” |  | Explores how social media anonymity fosters aggressive 'keyboard warrior' behaviour, illustrating pragmatics' role in context-driven language use, impacting meaning and interpretation. |
|  | **Impact on Mental Well-being and Anxiety**: Focuses on how social media usage relates to mental health issues like anxiety and depression. Prolonged exposure to social media can lead to increased anxiety, depression, and other mental health issues. | "This inhibits their ability to meet face to face and so they use social media for social contact even at a young age. We have seen that this brings numerous benefits. They are able to remain grounded by communicating with their peers. They collaborate over issues concerning them (including homework etc). They tell us that they value the ability to be in touch with someone else their age. However, there are also risks. We have seen safeguarding risks emanate into threats and real concerns that increase anxiety and impact negatively upon mental health. This is incredibly worrying for children and their families. We also note that some 'trends' in behaviour are influenced by social media. This also impacts negatively upon mental health as it changes attitudes and perceptions. Pupils feel that they must do something influenced by social media even if they know that this isn't right. This results in an internal moral conflict increasing anxiety and impacting upon mental health." |  | Delves into the intricate link between social media and mental health, examining changes in communication patterns, attitudes, and perceptions. It underscores the impact of social media on behaviour and moral conflicts, pertinent to the study of pragmatics in linguistics, revealing how language use and communication norms can affect mental well-being and anxiety. |
|  |  | " Depending on the individual child, their household, parent input and usage - it's hard to determine an exact answer. Though with current rates of self harm behaviours from young people, perhaps a “big influence on their mental health. |  | Highlights how language and communication on social media influence children's mental health. It emphasises the impact of social media content on their well-being. |
|  | **Always-On Culture and Lack of Downtime**: This theme highlights the absence of breaks from social interaction due to constant social media connectivity. Social media's pervasive nature means children are continually engaged, leading to a lack of rest and downtime. | "There's also a significant issue with FOMO (Fear of Missing Out) where young individuals are using social media late into the night because they worry about missing out on events or updates despite our efforts to educate them on safe usage and our frequent communication with parents. Additionally, we've seen a substantial increase in bullying especially since the onset of the COVID-19 pandemic. Much of this bullying occurs on social media during the evening and late at night. Regrettably, we find ourselves addressing these issues during the day as parents often don't want to take responsibility for them during the nighttime hours.” |  | Explores social media's language of inclusion and exclusion, highlighting FOMO's linguistic aspect in creating urgency and anxiety, impacting mental health through constant engagement via messages and posts. |
|  |  | “Children think that they must be on social media in order to be part of a social group which means more children are having phones at an early age […….] But, for me, the biggest problem is never having any down time away from social media - I think that must be very difficult for young people. |  | Reveals how social media extends and intensifies peer interactions, fostering a constant connectivity culture that linguistically influences how children communicate. It also touches on linguistic norms in online communication, affecting children's downtime and rest. |
|  | **Disruption of Real-World Interactions**: Concerns the way social media replaces or diminishes face-to-face interactions. Over-reliance on digital communication can hinder the development of real-world social skills and interactions. | "It is difficult enough for children to navigate the social etiquette of friendship without the issues [*named social media*]. Reliance on social media develops a need for acceptance by peers and creates questions about am I likeable do I fit in am I big small fashionable unfashionable. It stops children enjoying childhood" |  | Signifies a linguistic shift in social interactions due to digital reliance, impacting conversational and interpersonal skills usually developed through face-to-face interactions. Digital media may hinder natural linguistic and social competency growth. |
|  |  | "Social media portrays an element of people's lives. It affects children and young people because it can reduce their self-esteem. Forgetting that the pictures and videos have been carefully constructed and 'real' can be damaging. People can feel like they are less than they are. People compare themselves to what they see online" |  | Highlights identity and self-expression construction. Social media's altered reality can distort understanding of human interaction and self-expression, affecting authentic, spontaneous language use as focus shifts to online personas. |
| Social Work | **Influence on Self-Perception and Body Image**: This theme relates to how social media affects children's self-esteem and body image. Many headteachers observed that children compare themselves to unrealistic standards seen on social media, which can lead to issues with self-confidence and self-worth | "Self-esteem and body image are adversely affected by the unrealistic expectations portrayed on social media. This phenomenon is observed equally in boys and girls." |  | Gocusing on social media's impact on children's self-esteem and body image, emphasising the negative effects of unrealistic expectations. It underscores the universality of this issue across genders. |
|  |  | "Social media portrays an element of people's lives. It affects children and young people because it can reduce their self-esteem. Forgetting that the pictures and videos have been carefully constructed and 'real' can be damaging. People can feel like they are less than they are. People compare themselves to what they see online." |  | Highlighting social comparison's role in shaping self-perception and body image via curated social media content. It underscores the impact on young individuals' well-being and informs social workers' efforts to address insecurities and self-image problems. |
|  | **Online Bullying and Peer Pressure**: This theme highlights the prevalence of cyberbullying and peer pressure facilitated through social media platforms. It is noted that social media extends the reach and intensity of bullying outside of school settings. | "Social media has a significantly detrimental effect on children's mental health. We see constant pressures from social media in terms of how children feel they need to look and act. In addition, bullying and peer pressure via social media is a constant battle and we are always having to deal with issues between children in school based on something that has happened on social media." |  | Addressing cyberbullying and peer pressure, extending beyond schools to social media, affecting children's mental health. It underscores the need for adapted interventions and strategies. |
|  |  | "Additionally we've seen a substantial increase in bullying especially since the onset of the COVID-19 pandemic. Much of this bullying occurs on social media during the evening and late at night." |  | Highlighting the rise in online bullying, notably during the COVID-19 pandemic. The focus on evening and night incidents underscores the continuous, intrusive nature of cyberbullying, necessitating interventions addressing its 24/7 impact on children's well-being. |
|  | **Impact on Mental Health and Well-being**: Many comments emphasise the negative impact of social media on children's mental health, including anxiety, depression, and other emotional disturbances. | “The use of social media, without the appropriate support, monitoring, education can have a detrimental impact on children's mental health. We have seen examples of eating disorders perpetuated by overuse of Insta (as an example), as well as incidents of cyber bullying that children are subjected to 24/7.” |  | Emphasising the daunting challenge of addressing social media's pervasive influence on children's well-being and the need for educational efforts to mitigate associated risks. |
|  |  | “Children and young people rely heavily on social media for information, contact with friends, networking etc. A perfect world is often portrayed through social media and children and young people can be drawn into thinking their life should be like that, they should look perfect etc etc. Sometimes reality is lost, and they are left feeling as though they don't conform, don't fit in, are inferior to others etc. Self-esteem can be damaged. Long hours spent staring at phones or tablets whilst interacting with social media also affects mental health and well-being as they spend less time socialising in person, more time indoors, less time communicating with others properly etc” |  | Highlighting social media's adverse effects on children's mental health and self-esteem, emphasising unrealistic standards and social pressures, vital for addressing the holistic well-being of vulnerable children and young people influenced by social media. |
|  | **Addictive Nature and Disruption of Social Skills**: The addictive quality of social media and its impact on social skills and real-life interactions is a recurring theme. Headteachers express concern about the over-reliance on digital communication at the expense of face-to-face interactions. | " The children appear to be in constant touch with each other which means that they get no down time from each other or school. I feel that it is making their world shrink a little!” |  | This quote is relevant from a Social Work academic perspective as it addresses the addictive nature of social media and its impact on children's social interactions. The addictive quality, indicated by the extensive hours spent on social media. |
|  |  | " Childhood seems to be reducing with many children 'addicted' to their phones. I think that for many children it is modelled at home, with parents and older sibling sharing content and using phones- games, social media as a "baby sitter" = keeps the children quiet. Children now have far more sexualised behaviours which I think can be linked to social media.” |  | Emphasises social media's addictive influence on children, its role in disrupting social development, and its potential for negative behaviours, underscoring the need for monitoring and limits. |
|  | **Altered Communication Dynamics and Family Influence**: This theme covers how social media changes communication styles and dynamics, not just among peers but also within family settings. There's a concern about the influence of parents' social media use on children. | " It reduces focus and conditions them for over stimulus. It is impacting on their ability to self-regulate or co-regulate, due to how much it is used by parents to 'quieten' their much younger children when they are dysregulated. It is impacting upon self-image and forcing an unhealthy comparison with too many different people and too many unrealistic presentations of the lives of others.” |  | Highlights the impact of social media on children's self-regulation, parenting styles, unrealistic standards, and their overall emotional development, crucial in social work for fostering healthy communication and self-esteem. |
|  |  | "I believe that there is a major social change underway in terms of parenting style and that children are not only affected by their own use of social media (e.g., our 10-11 year olds who all have phones but are simply not mature enough to use WhatsApp without being unkind to each other) but by the social media use of their parents. I think we should look at parental use of social media and how it impacts on children as well as children's own use of social media. Every school has a toxic parent WhatsApp group or two and this negatively affects the children. When children are aware that parents don't value or respect a teacher the child can disengage with learning - and that in turn affects confidence self-esteem. I also wonder that parents spend so much time on their phones that they don't spend as much time with their children. Many children with behavioural or MH issues these past two years have said 'Oh my mum is always on her phone'" |  | Highlights the influence of social media on parenting styles, including toxic Social Media groups, affecting children's behaviour and family dynamics. It's crucial for social work and understanding parental impact. |
| Anthropology | **Cultural Norms and Expectations**: This theme delves into how social media shapes and reinforces societal expectations and norms. Headteachers frequently mention the pressure social media places on young people to conform to certain standards of appearance and behaviour, which often leads to negative self-perception and mental health issues. | "Young people are impressionable and often have a strong desire to be involved and part of something popular. They rarely wish to be an outsider unpopular or a target for bullies. This pressure is unhealthy and often expectations are unrealistic. |  | Emphasises cultural socialisation through peer influence, highlighting the human need for social acceptance. It also delves into group dynamics, social stratification, and cultural constructs of popularity. |
|  |  | “Social media does impact upon children’s mental health as they are constantly exposed to the ‘ideals’ and an untrue representation of life where they only see the positive parts of influencers lives. They also are exposed to dangerous habits which could impact upon their health such as vaping. They also develop an opinion of an ‘ideal’ body and then judge themselves against this when the reality is these pictures and clips have been edited.” |  | Shapes cultural norms and values, influences global exchange, explores representation vs. reality, and impacts health and cultural practices. |
|  | **Social Connectivity and Isolation**: This theme explores the paradox of social media as a tool for both connection and isolation. While social media can foster relationships and a sense of belonging, it can also lead to feelings of loneliness and exclusion. | They feel like their are standards to live up to - busy social lives perfect rooms and decor expensive phones and clothes etc. That life is happy and wonderful. There's a lot of pressure to 'fit in' |  | Highlights material culture, where objects symbolise identity. It also explores social connectivity's portrayal as success and constructs of happiness, significant in anthropology for understanding social stratification and cultural ideals. |
|  | . | Some young people put a lot of stock into how many friends/followers and likes and comments they get. So low numbers or negative comments can damage self-esteem and views about their own self worth" |  | Highlights how digital social networks influence self-perception and cultural values, showcasing the quantification of social connections and the paradox of connectivity and isolation, relevant in anthropology. |
| Health Sciences | **Digital Interaction and Communication**: This theme focuses on how digital communication on social media platforms alters traditional forms of interaction and language. It examines the impact of online communication on the mental health and social skills of young individuals. | "It reduces focus and conditions them for over stimulus. It is impacting on their ability to self-regulate or co-regulate due to how much it is used by parents to 'quieten' their much younger children when they are dysregulated. It is impacting upon self-image and forcing an unhealthy comparison with too many different people and too many unrealistic presentations of the lives of others." |  | Underscores the health sciences focus on digital media's psychological effects on children: overstimulation, impaired self-regulation, altered interactions, and negative impacts on cognitive and emotional development. |
|  |  | "Social Media has a lot of influence on children, young people and young adults alike. In children and young people especially it can and has had an impact on their mental health in an extremely negative way and I have seen this first hand. Working in a Primary School where children as young as seven have been able to access social media has been quite frightening. We have had incidents of self harming, school refusers, children talking online to unknown strangers, and depression in children due to what they have seen and experienced online. I believe it is more prevalent than we think and the harm it can do can potentially stay with children for a long time." |  | Highlights the crucial mental health impacts of digital interaction on children, including self-harm and depression, emphasising the need for health sciences to study the psychological implications of intense social media exposure. It underscores the transformation of traditional social interactions and the severity of online experiences on children's mental health and social skills. |
|  | **Social Comparison and Self-esteem**: Comments reflect concerns about children comparing themselves to the often unrealistic standards seen on social media, affecting their self-esteem. | " Young people are exposed to many harmful things that they would otherwise not be seeing, and they believe images they see are the expected norm. This puts pressure on them to live up to what they think they should look like, do, behave like etc. when they can’t live up to that expectation they believe they have a deficit and without support they can’t overcome this. It is unrealistic and creates pressure on young people, which leads to low self esteem and confidence.” |  | Emphasising the psychological impact of social media's unrealistic standards on young people's self-esteem and self-worth due to social comparisons. |
|  |  | "I think it has a detrimental effect on children young people and young adults. It can cause self-doubt low self esteem. It can be a platform for bullying shaming etc. I also think young people are becoming addicted to social media and are not interacting face to face as much." |  | Underscores social media's negative impact on young peoples’ mental health, including self-doubt, low self-esteem, bullying, and addiction. It aligns with health sciences' focus on these mental health and social development issues. |
|  | **Cyberbullying and Peer Pressure**: Several responses indicate that social media can be a platform for bullying and peer pressure, significantly impacting mental health. | "To a large extent. Children and young people are no longer able to step away from peer pressure. Previously bullying and peer pressure were situations which mainly occurred at school or when with friendship groups in social situations. Now with high access to phones and social media the pressure is more present than ever. Make up videos, fitness videos, videos from influencers who suggest certain types of behaviour are constant and children and young people's mental health is in crisis because home is no longer the safe space it was." |  | Underscores the pervasive nature of peer pressure and bullying on social media, a concern in health sciences, with a focus on mental health and developmental health. |
|  |  | "Like anything there is both good and bad. It can help forming relationships, connections with others with shared interests and be a source of entertainment. Negatively there are a lot of people who use it to bully/target others, make judgments, or present unrealistic expectations, create shame and comparisons." |  | Highlights social media's dual nature, particularly its mental health impact on cyberbullying and unrealistic expectations, crucial topics in health sciences concerning children and adolescents' well-being. |
|  | **Impact on Physical Health and Lifestyle**: The influence of social media on physical health behaviours, like eating disorders and physical activity levels. | "In my opinion I believe social media plays a huge role in the influence of what the child thinks and feels about themselves. Body image and the social media sites which influence the negative thoughts and feelings." |  | Underscores the connection between social media's influence on body image and its potential to lead to health issues like eating disorders and negative self-perception. It highlights the importance of addressing these factors in promoting healthy lifestyles among children. |
|  |  | "I have seen it cause a number of eating disorders across the ages as well as hiding away and causing depression. The vast majority of social media makes children feel inferior/angry/misinformed/exhausted." |  | Underscores the direct link between social media, eating disorders, and mental health issues, vital concerns in health sciences due to their significant impact on physical and psychological well-being among young people. |
|  | **Parental Influence and Modelling**: The role of parental behaviour and its impact on children's social media use is highlighted, suggesting that children's habits are influenced by their parents' use of social media | "Change underway in terms of parenting style and that children are not only affected by their own use of social media (e.g., our 10-11 year olds who all have phones but are simply not mature enough to use WhatsApp without being unkind to each other) but by the social media use of their parents. I think we should look at parental use of social media and how it impacts on children as well as children's own use of social media. Every school has a toxic parent WhatsApp group or two and this negatively affects the children. When children are aware that parents don't value or respect a teacher the child can disengage with learning - and that in turn affects confidence self-esteem. I also wonder that parents spend so much time on their phones that they don't spend as much time with their children. Many children with behavioural or MH issues these past two years have said 'Oh my mum is always on her phone'." |  | Highlighting how parental behaviour on social media can impact children's attitudes, behaviour, and mental health outcomes, emphasising the interconnectedness of family dynamics and children's well-being. |
|  |  | "Sometimes it feels in schools we are dealing with fallout from social media outside of school. Parents who themselves are prey to social media do not seem to recognise the harm it does to their children unless properly supervised. I think the internet and social communication has been a game changer and has opened up so many opportunities to our children but we must also be very mindful of the destructive nature it can have. Responses on social media often show a lack of regulation by children quick to respond in aggressive tones causing extreme hurt. This lack of regulation impacts themselves and others. |  | This quote underscores the health sciences' concern for children's mental health and behaviour affected by social media, emphasising parental supervision's role in mediating these effects and the importance of parental involvement. |
