# Supplementary material for: Synthesizing perspectives: Crafting an Interdisciplinary view of social media’s impact on young people’s mental health
Source: PLoS One. 2024 Jul 15;19(7):e0307164. doi: 10.1371/journal.pone.0307164 (PMC11249244; doi:10.1371/journal.pone.0307164)
Supplement: S5 Table — (DOCX) [file pone.0307164.s005.docx]

**S5 Table. Thematic Analysis of Secondary School Headteacher Narratives around Social Media effects on young people’s mental health.**

| Academic Domain | Theme | Quote |  | Rationale |
| --- | --- | --- | --- | --- |
| Psychological | **Anxiety and Stress**: Social media often leads to increased anxiety and stress among young people due to constant exposure to negative or overwhelming information. This is aligned with clinical psychology, focusing on mental disorders like anxiety. | "Young people are very much influenced by trying to fit in and be accepted by others. Social media gives them a false expectation of what 'real' people look like and places enormous pressures on them to live an '[*named social media*] worthy' life. This is a totally unsustainable pressure and leads to young people suffering from anxiety and extremely high stress levels" |  | Delves into the psychological aspect of social comparison theory, revealing how social media fosters unrealistic comparisons, causing feelings of inadequacy, anxiety, and the pressure to conform, intensifying stress. |
|  |  | "Social media affects children's young people and young adults mental health negatively by limiting direct contact with peers and encouraging constant comparison online which often leads to low self-esteem anxiety and depression. This is clearly evident across my school” |  | Supports clinical psychology by highlighting social media's link to anxiety and depression, emphasising reduced face-to-face interactions and online comparisons affecting self-esteem, crucial for understanding psychosocial aspects of stress in young people. |
|  | **Self-Esteem and Body Image Issues**: Social media creates unrealistic expectations about appearance and lifestyle, which can negatively affect self-esteem and body image. This theme is related to health psychology, which explores psychological factors in health and illness. | “In the young people I teach, it seems to be the largest factor in causing mental health problems. I teach at a girls' school, and social media causes body image problems as well as causing low self-esteem" |  | Aligns with health psychology, highlighting social media's role in distorting body image perceptions, contributing to self-esteem issues, and emphasising the field's focus on societal influences on mental health and self-perception. |
|  |  | "I think social media use in these groups has an overall negative impact on mental health as they are constantly bombarded with information which demonstrates unrealistic beauty and achievement standards at a relatively young age when they have not learned to evaluate it as such" |  | Underscores health psychology's concern with unrealistic social media standards affecting young people's self-esteem and body image, emphasising their vulnerability and the field's examination of societal influences on mental health. |
|  | **Social Skills and Real-World Interaction**: Overuse of social media can diminish face-to-face communication skills and real-world interaction, which is a concern in developmental psychology. | “… many young people spend a lot of their time on social media which can affect their communication with people in real life or affect their sleep patterns which can have a detrimental effect on their mental health... Many young people only communicate through socials and often lose confidence and soft skills when talking 'in real life'" |  | Highlights developmental psychology's concern over excessive social media hindering real-life communication skills and confidence. It underscores how it erodes 'soft skills,' crucial for social development. |
|  |  | "Social media has been a fantastic way for young people to embark on an upward trajectory to entrepreneurship through influencing. However, it can become addictive and have a negative impact on allowing people to develop social skills in real life" |  | Highlights the dichotomy of social media's influence on social skills, acknowledging its potential for skill development yet emphasising its addictive nature and potential hindrance to healthy development in developmental psychology terms. |
|  | **Peer Influence and Cyberbullying**: The influence of peers and the prevalence of cyberbullying on social media can have profound effects on mental health, relevant to social psychology. | "Social media use among these age groups has an intense influence on their mental health. In general kids particularly younger ones have a deep need to belong socially and being able to conform to group ideals - or not being able to - can profoundly affect a social media user’s self image which in turn is inextricably linked with mental health. Social media can intensify the formation of both in-groups and out-groups and children and adolescents who find themselves in the latter in particular are especially vulnerable to negative mental health effects from cyberbullying and lack of self esteem" |  | Underscores peer influence on young social media users, touching on social psychology's core themes of belonging, conformity, in-groups, out-groups, and their effects on mental health. It aligns with research in social psychology on peer influence and cyberbullying. |
|  |  | "I see cyberbullying as one of the biggest threats. Children can create so much drama in their group chats gossiping and being rude to each other whilst chasing 'likes' that many of them end up feeling excluded, anxious and unable to cope with the negative comments" |  | Delving into cyberbullying's mental health impact on children in group chats, emphasising social validation and peer behaviour, aligning with social psychology's focus on online interactions and their effects on well-being. |
| Sociological | **Influence on Self-Perception and Identity Formation**: This theme explores how social media shapes young people's perceptions of themselves and their identities. It includes concerns about unrealistic beauty standards and the pressure to conform to certain lifestyles, leading to issues like low self-esteem and body image disorders. | "Social media has a massive influence on them. Children and young people are far more conscious of their appearance now and as a result are wearing make up much more younger (females) or are spending time in the gym building muscles (males) compared to 20 years ago" |  | Explores social media's sociological impact on young peoples’ self-perception and identity formation. It emphasises the significance of 'likes' and appearance, reflecting conformity to unrealistic beauty and lifestyle standards. |
|  |  | "Self-esteem is one big area in that young people are developing their self-image in apps that encourage filters. Addiction is another- the pursuit of likes and engagement providing a temporary high of sorts plus fear of missing out and not responding to / viewing notifications. I think social media interactions impact organic friendship development and facilitate superficial relationships that leave young people feeling connected but lonely" |  | Explores social media's impact on young peoples’ self-esteem, self-image, and identity. Filters create unrealistic beauty standards, while 'likes' addiction affects genuine social connections, leading to loneliness. |
|  | **Social Interaction and Community Building**: This theme addresses how social media either fosters or hinders social interactions and community building among young people. It includes the role of social media in creating online communities and its effect on face-to-face communication skills. | "I feel there's positive influences through connection to others and having a sense of community. It also provides a means of self-expression and access to information and support. However, I think overuse can be a negative thing and perhaps lead to anxiety" |  | Highlighting social media's dual role in fostering connections and community while potentially hindering real-life social interactions, essential for understanding its impact on modern society. |
|  |  | "… young people we look after... are addicted to these apps and sites that have been designed to attract their attention and are powerful enough to cut them off from 'real interactions'. Friendships and communication has rapidly changed with young people as these sites grow in popularity and abundance" |  | Exploring how social media transforms traditional social interaction and friendship development among young people. It highlights addiction, reduced face-to-face interactions, and changing social dynamics. Crucial for studying technology's impact on human behaviour and social structures. |
|  | **Mental Health and Well-being**: This theme focuses on the direct impact of social media on mental health, including issues like anxiety, depression, and stress. It also looks at how social media usage can be both a positive and negative influence on mental well-being. | "It feeds into negative thoughts regarding their self including self-worth & body-image by creating an unrealistic portrayal of other people. I think this leads to a cycle of comparison which is enlarged compared to real life where this may only happen with a select number of people they know friends etc whereas they are looking at a large number of people and the sheer amount is overwhelming" |  | Exploring social media's impact on mental health, emphasising unrealistic portrayals, harmful comparisons, and their role in anxiety, depression, and stress, crucial for understanding the social media-mental health nexus. |
|  |  | "Sometimes it can be a healthy source of connection allowing them to keep in touch with their friends and stay involved with things they find interesting. But more often it seems to contribute to feelings of anxiety, self-consciousness, and depression" |  | Balancing social media's positive and negative mental health effects, highlighting anxiety, self-consciousness, and depression, crucial for comprehending its intricate influence on young peoples’ well-being. |
| Education Studies | **Digital Literacy and Curriculum Development**: This theme involves integrating digital literacy into the curriculum to help students navigate social media healthily. It aligns with Curriculum and Instruction, focusing on developing educational programs that incorporate contemporary challenges like social media usage. | "However social media also provides connection and creativity. As a mom and teacher staying involved and teaching digital literacy are key. Collaborating with others helps create a balanced approach to supporting mental health in the digital age" |  | Underscores the need for educators to prioritise digital literacy to guide students in navigating social media's dual potential for creativity and harm and collaboration in curriculum development is essential. |
|  |  | "The only thing that I have found that works is to also teach about teenage brain development alongside teaching about how technology and social media affects an individual" |  | Stresses the importance of integrating education on technology's physiological and psychological effects, advocating for a comprehensive approach that addresses both technical and developmental aspects in curriculum development. |
|  | **Psychosocial Impact of Social Media**: Related to Educational Psychology, this theme reflects the psychological effects of social media on students, like self-esteem issues and stress, and the need for educational interventions to address them. | “Social media has a huge impact on young people's mental health. They only see the highlight reel of people's lives and a highly edited version of reality eg edited pictures from influencers. this has a negative impact on body image and general mental health. there is also no limit to what they can access they can be subjected to online bullying. suicide rate particularly in young women has increased since [*named social media*]" |  | Delves into the psychological impact of social media on young people, highlighting body image distortion, mental health challenges, and rising suicide rates among young women. It aligns with educational psychology's need for interventions. |
|  |  | "It has a significant negative impact. It destroys self esteem and encourages narcissistic thoughts. It causes young people to disconnect from the real world and real situations. It causes them to lack basic social skills" |  | Highlights social media's adverse psychological effects, including reduced self-esteem, narcissism, and impaired social skills. It underscores the importance of targeted educational psychology interventions for healthier student development. |
|  | **Inclusive Digital Practices for Social, Emotional and Mental Health Needs Education**: In the realm of Special Education, this theme focuses on tailoring social media education to the needs of students with disabilities, ensuring they are not left vulnerable to the negative aspects of digital life. | “Role modelling is a good example and seeing oneself through alike social reference groups portrayed in a positive way can support young people to develop a more positive self esteem. role modelling through social model has greater reach and connects people in a much wider way then would otherwise be possible and is particularly relevant to those who feel marginalised. This connectivity leads on to greater opportunities for not only accessible and augmentative communication but also for networking positively with others and engaging with protective factors and supportive interventions to prevent a decline in mental health"​​ |  | Explores social media as a tool for positive role modelling, especially for marginalised young people. It underscores the significance of inclusive digital practices for students with disabilities and those seeking connection and support. |
|  |  | "I think social media can have a positive influence on the mental health of marginalised young people to help them feel less alone. For example, a transgender young person might meet others online who will make them realise they aren't alone. However, this can also have a negative effect if these people are the wrong kind of influence or pose a threat to the vulnerable young person. I think social media needs to be monitored for young people to help them from struggling with the mental health effects" |  | Recognises social media's dual role for marginalised groups like transgender young people, offering community and risk. It emphasises inclusive digital practices, highlighting educators' role in guiding and safeguarding online experiences. |
|  | **Leadership in Digital Era**: Falling under Educational Leadership and Administration, this theme explores how school leaders can guide policies and practices around social media use and its impact on student well-being. | "Schools have a role to play in educating young people on the pitfalls of social media use and the importance of safeguarding their mental health and so do families"​ |  | Underscores schools' responsibility in educating about social media risks, advocating for policies prioritising student mental health. Collaboration between schools and families is crucial for effective leadership in the digital age. |
|  |  | "They talk about pressure these apps make them feel to look or think a certain way and I have found that all recommended teaching methods fail in helping them to use these sites with caution and moderated use. The only thing that I have found that works is to also teach about teenage brain development alongside teaching about how technology and social media affects an individual" |  | Highlights the complexities for school leaders dealing with social media's impact on students' mental health and self-perception. It advocates innovative, adaptive teaching methods focusing on psychology and brain development. |
|  | **Policy Development for Digital Well-being**: Within Educational Policy and Reform, this theme addresses the need for policies at school and broader levels to regulate social media use and protect students’ mental health. | "The use and overuse of social media is strongly negatively impacting the mental health of young people. I am becoming increasingly concerned for the mental welfare of the current school population unless something is done to regulate this industry" |  | Urgently calls for social media regulation to safeguard students' mental health. It underscores educational leaders and policymakers' responsibility to develop effective policies mitigating social media's adverse effects. |
|  |  | “I think social media can have a positive influence on the mental health of marginalised young people to help them feel less alone. For example, a transgender young person might meet others online who will make them realise they aren't alone. However, this can also have a negative effect if these people are the wrong kind of influence or pose a threat to the vulnerable young person. I think social media needs to be monitored for young people to help them from struggling with the mental health effects" |  | Acknowledges social media's dual impact, particularly for marginalised groups like transgender young people. It stresses monitoring for mental health protection and calls for nuanced, tailored policies to ensure a safe digital environment for all students, especially the vulnerable. |
|  | **Social Media as a Societal Mirror**: Under Sociology of Education, this theme looks at how social media reflects and amplifies societal issues like inequality, bullying, and peer pressure, impacting educational practices. | "Social media use among these age groups has an intense influence on their mental health. In general, kids, particularly younger ones, have a deep need to belong socially and being able to conform to group ideals - or not being able to - can profoundly affect a social media user’s self-image which in turn is inextricably linked with mental health. Social media can intensify the formation of both in-groups and out-groups and children and adolescents who find themselves in the latter in particular are especially vulnerable to negative mental health effects from cyberbullying and lack of self-esteem." |  | Deonstrates social media's reflection of societal issues, including belonging, in-group/out-group dynamics, and their mental health effects. It highlights the importance of understanding these dynamics in education. |
|  |  | "I think social media use impacts the mental health of young people hugely. They no longer see airbrushed images in magazines on a weekly basis, they consume these images daily, often many times a day. They compare their lives to those of influencers whose job it is to sell products to us and young people cannot keep up with this. This leads to poor self-esteem and confidence issues." |  | Discusses social media's influence on young people, emphasising exposure to unattainable ideals. It underscores the importance of teaching critical media literacy and addressing its impact on students' self-esteem and mental health. |
| Political Science | **Digital Governance and Policy (Public Administration and Policy)**: This theme explores how the use and regulation of social media among young populations intersect with public policy and governance structures. It focuses on the role of educational institutions and families in guiding responsible social media use and its implications for mental health. | "Schools have a role to play in educating young people on the pitfalls of social media use and the importance of safeguarding their mental health and so do families.” |  | Underscores the vital role of educational institutions and families in promoting responsible social media use. It aligns with Digital Governance and Policy, emphasising structured guidance, policy development, and public-private collaboration. |
|  |  | “We see social media as both a lifeline and a negative influence on young people's mental health within our care. It is how they stay connected to their peers and in our context of being a full boarding school it has eased communication with family and friends who may live the other side of the World. They use social media to manage the urge to harm themselves through apps or social media profiles that have been created by experts who have developed preventative help in a way that young people are far more likely to access.” |  | Examines social media's dual role in young people's mental health, emphasising its potential benefits and drawbacks. It highlights the importance of policies for harnessing benefits and mitigating harm within Digital Governance and Policy, a concern of public administration. |
|  | **Social Media as a Socio-Political Tool (Political Theory)**: This theme delves into the philosophical and ethical considerations of social media as a tool for shaping young minds and influencing societal norms and values. | "Social media can be a positive influence in the lives of young people but can also have a negative impact - particularly where young people are drawn to inappropriate content or where they spend too much time online and do not have healthy face-to-face relationships. Schools have a role to play in educating young people on the pitfalls of social media use and the importance of safeguarding their mental health and so do families" |  | Underscores the impact of social media on young people and the ethical responsibility of schools and families. It raises questions about technology's influence on societal norms and values, relevant to political theory. |
|  |  | "Social media use among these age groups has an intense influence on their mental health. In general kids particularly younger ones have a deep need to belong socially and being able to conform to group ideals - or not being able to - can profoundly affect a social media user’s self-image which in turn is inextricably linked with mental health. Social media can intensify the formation of both in-groups and out-groups and children and adolescents who find themselves in the latter in particular are especially vulnerable to negative mental health effects from cyberbullying and lack of self-esteem" |  | Delves into how social media influences young people's mental health and social dynamics, addressing political theory's concern with social structures, norms, and the ethical implications of technology. |
|  | **Globalisation of Mental Health Issues (International Relations)**: This aspect considers the global impact of social media on mental health, transcending national borders and cultural contexts, and emphasises the need for international cooperation in addressing these challenges. | "We see social media as both a lifeline and a negative influence on young people's mental health within our care. It is how they stay connected to their peers and in our context of being a full boarding school it has eased communication with family and friends who may live the other side of the World. They use social media to manage the urge to harm themselves through apps or social media profiles that have been created by experts who have developed preventative help in a way that young people are far more likely to access" |  | Illustrates how social media fosters global connectivity among young people, transcending borders, which has implications for International Relations. It also highlights international collaboration in mental health via digital tools. |
|  |  | "Examples of this negative impact on mental health can include pressure to behave, look, and act in a certain way that is not necessarily linked to healthy behaviours. A recent example of this is the myriad of [*named social media*] [*named social media*] cases that seem to have swept social media. Added to this, social media has become a means of interacting and communicating with others that has no time or distance limitations. This has meant bullying can occur at any point in time on any day. Coupled with this is the use of anonymity facilitating faceless interactions that can perpetuate bullying even further as well as perpetuating behaviours that wouldn't necessarily occur in the real world. This latter point of the 'real world' also becomes interesting as we start to blur boundaries of what is real and what is not and understanding the lived experience of many young people now follows focuses on online connections through social media rather than physical connectedness" |  | Highlights how social media transcends time and space, affecting global issues like bullying and behaviour change. It underscores the blurring of online and offline realities, impacting mental health and necessitating international cooperation. |
|  | **Economic Impacts of Social Media (Political Economy)**: This theme examines the intersection between social media use and economic factors, such as the influence of social media on young people's career aspirations and the commodification of social media platforms. | "I think that overuse of social media by children has come at the expense of healthy real life social interactions and I worry that children are not learning how to behave in real life anymore. It's too early to see what impact this has as social media is of this generation but I'm interested to see what adults are produced from a whole generation of children who have grown up with mediated interaction through social media. I also worry that young people are fed a curated view of an ideal lifestyle through the social media channels and will" |  | Addresses social media's impact on young people's career aspirations, influenced by curated views of success. It also discusses the commodification of online interactions, relevant to the political economy of digital space |
|  |  | "To a massive extent they become a lot more uncomfortable with themselves having been seeing all the 'successful' people on social media. They also join stupid challenges that might be damaging to their physical health. However, I think for some people it is also useful as they can make friends even they struggle to do so in real life (if they struggle with social anxiety etc.). So I am a bit double-sided on my opinion on whether social media is positive or negative" |  | Explores social media's impact on self-perception and economic behaviour, driven by exposure to 'successful' individuals. It underscores the significance of understanding these dynamics in Political Economy. Additionally, it delves into physical health challenges tied to online trends, which have broader economic implications, relevant to the field's study of digital media's influence on behaviour and societal outcomes. |
|  | **Comparative Analysis of Social Media Impact (Comparative Politics)**: This theme involves comparing the impact of social media on mental health across different cultural and national contexts, examining the variations in social media usage, regulation, and its consequences on young people. | I think there is enough data now to understand that young adults are overwhelmingly negatively impacted by social media. There is a rise in depression, anxiety and general lack of engagement with adult responsibilities and much of this can be pointed back to the influence of social media. In particular, I have noticed a strong uptick in negative self-comparison due to social media, in which students have been comparing themselves to the best of the best in terms of looks, fashion, etc and then feel paltry and small in their little school by comparison. This is just one tip of the iceberg on a very serious problem. |  | Addresses the global impact of social media on self-comparison, mental health, influencers, and attention span among young people. It suggests a need for cross-cultural regulation and policy intervention, relevant to comparative politics. |
|  |  | "We see social media as both a lifeline and a negative influence on young people's mental health within our care. It is how they stay connected to their peers and in our context of being a full boarding school it has eased communication with family and friends who may live the other side of the World. They use social media to manage the urge to harm themselves through apps or social media profiles that have been created by experts who have developed preventative help in a way that young people are far more likely to access" |  | Highlights the diverse roles of social media in global contexts, especially in boarding schools. It emphasises the importance of comparative analysis to understand its varying roles and impact, relevant to international relations and comparative politics. |
| Philosophy | **Digital Identity Formation**: This theme explores how online personas and social media interactions shape one’s self-concept and perception. It's related to metaphysics as it delves into the nature of 'self' in the digital realm. | "A lot of students base their identity off their 'profiles' and 'likes' - which is a shame as they often see it as a measure of their 'worth'. |  | Explores the philosophical implications of the digital age on the concept of 'self,' highlighting the impact of online personas and validation on identity formation. |
|  |  | "Children and young people often create a persona on social media which does not accurately represent their actual character, views, and values. This then creates a discord that is hard to maintain." |  | Delves into the philosophical question of authenticity, revealing a contrast between genuine self and online persona. It underscores the impact of digital spaces on identity exploration and questions of authenticity in a digital age. |
|  | **Information Perception and Influence**: Tied to epistemology, this theme considers how information on social media is perceived and how it influences beliefs and behaviours. | "Considering the rather impressionable nature of young people it could also influence their behaviours especially with reports of social contagion and copying of trends and things they experience on social media. It could also have positive influence when used the right way to give them a broader worldview, learn about new things, interact with people and broaden their social network to learn more about new places and things." |  | Raises epistemological questions about information perception in young minds. It addresses "social contagion" and trend adoption on social media, highlighting how it influences beliefs and behaviours, aligning with philosophical discussions on knowledge construction and external influences on reality perception. |
|  |  | "It seems like some young people are being 'taught' how to use social media carefully and responsibly by their parents or older siblings - they seem well aware that what you see online is often not the real deal and in those cases I think they use social media in a way that can actually benefit their mental health." |  | Delves into epistemology, exploring the distinction between reality and appearance in the context of young people's discernment on social media. Emphasises the role of critical thinking in shaping beliefs. |
|  | **Ethical Implications of Virtual Interactions**: Reflecting on ethics, this theme addresses the moral aspects of online behaviour, including cyberbullying and peer pressure. | "Added to this social media has become a means of interacting and communicating with others that has no time or distance limitations. This has meant bullying can occur at any point in time on any day. Coupled with this is the use of anonymity facilitating faceless interactions that can perpetuate bullying even further as well as perpetuating behaviours that wouldn't necessarily occur in the real world."​ |  | Delves into ethical dilemmas of virtual interactions, exposing the ethical challenges of online anonymity and boundary-less engagement, raising questions about morality and responsibility in digital spaces. |
|  |  | "Examples of this negative impact on mental health can include pressure to behave, look, and act in a certain way that is not necessarily linked to healthy behaviours. A recent example of this is the myriad of [*named social media*] cases that seem to have swept social media. Added to this, social media has become a means of interacting and communicating with others that has no time or distance limitations. This has meant bullying can occur at any point in time on any day. Coupled with this is the use of anonymity facilitating faceless interactions that can perpetuate bullying even further as well as perpetuating behaviours that wouldn't necessarily occur in the real world. This latter point of the 'real world' also becomes interesting as we start to blur boundaries of what is real and what is not and understanding the lived experience of many young people now follows focuses on online connections through social media rather than physical connectedness"​ |  | Explores the ethics of social media's influence on behaviour, self-perception, and mental health among young people. It raises concerns about conformity, anonymity, blurring realities, and the moral responsibility of platforms. |
|  | **Logical Reasoning in Digital Discourse**: This is about the logic applied or misapplied in interpreting social media content and forming arguments based on it. | "It is consuming and for some the most secure or only source of information or companionship. It is deskilling young people and removing communication and the ability to navigate healthy and relations. Equally it is becoming the source of research and the 'gospel according to' where other more balanced sources exist." |  | Underscores the importance of source reliability and logical reasoning in the digital era. It delves into epistemology, highlighting the need for critical evaluation amid social media's dominance. |
|  |  | "Social media presents unrealistic filtered images and information which young people often take as real situations. It gives them unrealistic expectations and ideals of what they ought to do/be/like." |  | Examines the distortion of reality on social media and its effects on young peoples’ perception and logical reasoning, revealing a concern for philosophical inquiries into belief formation and critical thinking. |
|  | **Digital Aesthetics and Self-Representation**: A theme related to aesthetics, focusing on how beauty and art are portrayed on social media and their impact on self-image | "You can see these youngsters especially girls comparing themselves to and aspiring to what they see on [*named social media*] etc and having totally unrealistic beliefs and expectations which can be debilitating to their self-esteem and their mental health." |  | Delves into the influence of digital aesthetics, particularly on young girls' self-representation. It raises aesthetic and ethical questions about beauty, self-perception, and mental health in the digital age. |
|  |  | "Social media has a huge impact on young people's mental health. They only see the highlight reel of people's lives and a highly edited version of reality e.g., edited pictures from influencers. This has a negative impact on body image and general mental health. |  | This quote explores how curated social media images distort reality and influence aesthetics. It touches on philosophical inquiries into reality, representation, and beauty while raising ethical concerns about altered images' impact on self-perception. |
|  | **Governance and Social Structures Online**: Echoing political philosophy, this theme examines the role of social media in shaping societal norms and structures. | "… offline negative behaviour extends into the online world. However, I have also seen some fantastic benefits. Friends who have moved away can still communicate with each other, support for peers when they are having a difficult time at home. Entrepreneurial children using channels to start businesses. Creative use of YouTube channels to showcase musical talent or gaming prowess. In these cases where children are not necessarily prominent in the offline world they take on a new confident role online." |  | Explores the online world's reflection and transformation of offline social structures. It raises philosophical questions about online governance, societal norms, empowerment, and reshaping social hierarchies. |
|  |  | "Added to this social media has become a means of interacting and communicating with others that has no time or distance limitations. This has meant bullying can occur at any point in time on any day. Coupled with this is the use of anonymity facilitating faceless interactions that can perpetuate bullying even further as well as perpetuating behaviours that wouldn't necessarily occur in the real world."​ |  | Delves into the transformation of social norms and interactions in the digital realm, particularly regarding the effects of online bullying and anonymity. It's pertinent to political philosophy, raising questions about virtual space governance and ethical implications. |
| Media Studies | **Digital Influence on Identity Formation**: This theme explores how social media shapes the self-perception and identity of young individuals. It reflects the media theory aspect of understanding the impact of digital platforms on personal development. | "It affects their self image and causes them to become more judgemental of themselves and each other. It minimises the importance of human connection and instead creates self worth based on quantity (of followers) not quality"​ |  | Underscores how social media affects self-perception in young people, prioritising follower quantity over meaningful connections. It aligns with media studies theories on digital identity and personal development. |
|  |  | "I think it has a significant impact on mental health and well-being. Self-esteem is one big area in that young people are developing their self image in apps that encourage filters. Addiction is another- the pursuit of likes and engagement providing a temporary high of sorts plus fear of missing out and not responding to / viewing notifications. I think social media interactions impact organic friendship development and facilitate superficial relationships that leave young people feeling connected but lonely” |  | Highlights the intricate role of social media in shaping self-image, social interactions, and personal development among young individuals. It aligns with media studies theories on digital identity and social skills. |
|  | **Virtual Socialisation and Communication Dynamics**: This focuses on the shift from traditional face-to-face interactions to digital communication, highlighting the changes in social skills and interaction patterns among young people. | "… many young people only communicate through socials and often lose confidence and soft skills when talking 'in real life'. Also many young people have misconceptions on attitudes about various things as often opinions or differences of opinions can often be misunderstood when just in a text or meme etc. So as good as social media can be in some cases I think it's had a massive impact on young people and their understanding about the world in general” |  | Underscores the transformative impact of social media on communication, revealing a shift towards digital interactions, leading to challenges in interpreting non-verbal cues. It aligns |
|  |  | "It takes them away from the real world of social interaction and takes them into a world that is isolated. Social media can involve bullying, grooming, and pressures from peers" |  | Addresses the transition from in-person to digital social interactions, highlighting issues like bullying, grooming, and peer pressure in online spaces. It's relevant to Media Studies' examination of digital media's impact on social interactions and young peoples’ communication patterns. |
|  | **Media Literacy and Critical Consumption**: Reflecting on how young individuals interpret and engage with content on social media, this theme ties into the importance of understanding media messages and their impact. | "I truly believe social media if used correctly can have a positive impact on people however I do also believe that the effect it has on an individual can be detrimental to their mental health. It's not uncommon to see videos and photos of horrific scenes without searching for them as social media platforms push forward popular videos to the algorithm and many times these are traumatic videos. This makes young people's minds exposed to difficult things which subconsciously becomes normalised for them. Also I feel there is a serious problem with people aspiring to look like what they see on social media which gives them unrealistic expectations of society and can be damaging to their mental health" |  | Underscores the importance of media literacy, highlighting the subconscious normalisation of traumatic content and the impact of unrealistic societal expectations on social media. It's crucial in Media Studies' examination of media's influence and the need for critical consumption skills. |
|  |  | "They are addicted to these apps and sites that have been designed to attract their attention and are powerful enough to cut them off from 'real interactions'. Friendships and communication has rapidly changed with young people as these sites grow in popularity and abundance. [...] They talk about pressure these apps make them feel to look or think a certain way and I have found that all recommended teaching methods fail in helping them to use these sites with caution and moderated use" |  | Underscores the challenges of the digital world for young people, highlighting social media's addictive nature, influence on self-perception, and pressure to conform. It emphasises the importance of media literacy education in Media Studies. |
|  | **Online Community and Support Systems**: This theme explores how social media platforms can offer support and a sense of belonging, especially for marginalised groups, aligning with audience studies in media. | "offline negative behaviour extends into the online world. However I have also seen some fantastic benefits. Friends who have moved away can still communicate with each other, support for peers when they are having a difficult time at home. Entrepreneurial children using channels to start businesses. Creative use of YouTube channels to showcase musical talent or gaming prowess. In these cases where children are not necessarily prominent in the offline world they take on a new confident role online." |  | Underscores the positive aspects of social media, emphasising its role in creating and sustaining online communities, supporting distant friendships, aiding personal challenges, and promoting self-expression and entrepreneurship. It aligns with media studies' focus on digital empowerment and community engagement. |
|  |  | "I truly believe social media if used correctly can have a positive impact on people. [...] I feel there's positive influences through connection to others and having a sense of community. It also provides a means of self expression and access to information and support." |  | Highlights the ambivalent impact of social media on mental health, focusing on its positive aspects like fostering connections, self-expression, and support networks. It aligns with Media Studies, emphasising the role of online communities for marginalised individuals and audience studies. |
|  | **Media-Induced Psychological Impacts**: Focusing on the psychological effects of social media, such as anxiety, self-esteem issues, and stress, this theme directly correlates with media's influence on mental health. | "In general social media contributes to a lack of concentration and a shortening of the attention span in people possibly leading to ADHD. There is also an unhealthy relationship with one's own public image and how one shows oneself to others since social media promotes showing off to others and being too open about one's private life. The younger the person is exposed to social media the stronger the consequences on their mental health." |  | Delves into the psychological effects of social media, including attention span and self-image issues. It underscores the concern in Media Studies about media's impact on mental health and self-esteem, especially among younger users. |
|  |  | "Examples of this negative impact on mental health can include pressure to behave, look, and act in a certain way that is not necessarily linked to healthy behaviours. A recent example of this is the myriad of [*named social media*] cases that seem to have swept social media. Added to this, social media has become a means of interacting and communicating with others that has no time or distance limitations. This has meant bullying can occur at any point in time on any day. Coupled with this is the use of anonymity facilitating faceless interactions that can perpetuate bullying even further as well as perpetuating behaviours that wouldn't necessarily occur in the real world. This latter point of the 'real world' also becomes interesting as we start to blur boundaries of what is real and what is not and understanding the lived experience of many young people now follows focuses on online connections through social media rather than physical connectedness" |  | Addresses how social media influences self-perception and behaviour, emphasising media's impact on personal identity and norms. It also explores anonymity, boundary blurring, bullying, mental health, and changing social connections, central themes in media studies. |
| Linguistics | **Perceived Reality vs. Actual Reality**: This theme explores how social media creates a distorted reality that young people may perceive as true, leading to unrealistic expectations and comparisons | "Social media presents unrealistic filtered images and information which young people often take as real situations. It gives them unrealistic expectations and ideals of what they ought to do/be/like. And if a young person has low confidence or self esteem these posts can be the triggers which cause them to spiral down in their mental health"​ |  | Underscores how language and imagery on social media influence young people's perceptions, shaping self-identity and mental health. It relates to semiotics, narrative construction, and the dissonance between perceived and actual reality. |
|  |  | "There is always this need to be as good or look as good as friends. There is also the problem with children having so many 'friends' on Social Media and not really knowing most of them. There are many instances of bullying going on and children not knowing where or who to turn to for help. It can and does affect their mental health very much"​ |  | Highlights linguistic aspects in self-perception and social media. It explores comparative language, redefined friendships, communication challenges, narrative construction, and their effects on mental health, emphasising issues of bullying and |
|  | **Influence on Self-Image and Confidence**: This theme addresses the impact of social media on young people's self-esteem and body image. | "It feeds into negative thoughts regarding their self including self-worth & body- image by creating an unrealistic portrayal of other people. I think this leads to a cycle of comparison which is enlarged compared to real life where this may only happen with a select number of people they know friends etc whereas they are looking at a large number of people and the sheer amount is overwhelming." |  | Delves into how social media language and imagery distort reality, leading to harmful self-comparisons and unrealistic body image ideals among young people, highlighting linguistic mechanisms at play. |
|  |  | "Students have no 'switch off' time and live a great proportion of their lives through social media. A lot of students base their identity off their 'profiles' and 'likes' - which is a shame as they often see it as a measure of their 'worth'." |  | Highlights how social media language and metrics impact self-esteem, shaping self-concept through linguistic cues and social validation, emphasising the connection between language and self-image. |
|  | **Constant Connectivity and Mental Overload**: This theme involves the mental strain caused by the always-on nature of social media, leading to issues like anxiety, stress, and inability to disconnect. | "Social media has ruined communication - children do not know how to talk to each other properly now. Bullying online is a real concern. Group chats cause an inordinate amount of mental health concerns amongst the student population. There is no escape from unpleasantness. Addiction to media is also a huge concern. Wanting to feel the same as everyone else also causes issues. I have worked in education for 20 years and have seen the rise in mental health cases as part of my role. Most stem from something online." |  | Discusses how constant social media connectivity has altered communication norms and language use, leading to mental health issues and online bullying. |
|  |  | " It is 'always on' meaning that they never have a chance to switch off an move away resulting in hyperstimulation. It also offers an inexhaustible amount of information which is overwhelming and unhelpful." |  | Addresses the linguistic challenges of continuous social media connectivity, emphasising the overwhelming nature of information flow and its impact on language processing and mental health. |
|  | **Cyberbullying and Peer Pressure**: This theme highlights the role of social media in facilitating bullying and peer pressure, impacting mental health. | "To a great extent. I see cyberbullying as one of the biggest threats. Children can create so much drama in their group chats gossiping and being rude to each other whilst chasing 'likes' that many of them end up feeling excluded anxious and unable to cope with the negative comments." |  | Highlights how language is used in cyberbullying and peer pressure on social media, emphasising its impact on mental health and social dynamics. |
|  |  | "There is a constant pressure to conform to an image to an unrealistic physical profile and anyone who falls short can feel a failure. Normal disagreements between people can escalate into full-blown nasty wars with many others, often anonymous bystanders joining in. Anonymity is a huge issue as for many people they will say things that they would never say face to face." |  | Highlights how language exacerbates conflicts and mental health issues on social media, emphasising the amplification of disagreements and the role of anonymity in aggressive language use. It reflects the linguistic dynamics of online communication, particularly in cyberbullying and peer pressure research. |
|  | **Positive vs. Negative Influence**: Reflecting on the dual nature of social media, this theme captures how it can be both a positive tool for connection and a negative source of distress. | "Social media use can have both a positive and negative influence on people's mental health. For those living in isolated areas it can be a great way of keeping in touch with friends and family which would have a positive impact. However, it is easy for young people to be exposed to cyberbullying, inappropriate media content, and the pressure of trying to live up to the unrealistic expectations of so-called influencers. All of this can negatively impact their mental health." |  | Highlights the dual influence of social media on mental health through language. It discusses the positive aspect of maintaining connections and the negative aspect of cyberbullying and unrealistic expectations. Linguistic elements play a crucial role in shaping users' perceptions and experiences on social media. |
|  |  | "Social media has the potential to be used as a positive influence with children and young people raising awareness of mental health, body positivity, etc., but that would require them to follow positive influences. However, more often than not, they follow friends/celebrities and end up in a cycle of comparison which has a negative impact on their mental health." |  | Underscores how language and content on social media can positively promote mental health awareness or negatively contribute to harmful comparisons and distress. |
| Social Work | **Social Skills and Interpersonal Relationships**: This theme focuses on how social media affects young people's ability to form and maintain healthy interpersonal relationships. The headteachers observe a decline in face-to-face communication skills and an increase in online interactions. | "Social media can be a positive influence in the lives of young people but can also have a negative impact - particularly where young people are drawn to inappropriate content or where they spend too much time on line and do not have heathy face to face relationships. Schools have a role to play in educating young people on the pitfalls of social media use and the importance of safeguarding their mental health and so do families." |  | Recognises the impact of social media on social skills and relationships among young people, emphasising the importance of direct human interactions and the role of social work in intervention and education for balanced social development. |
|  |  | "Many young people spend a lot of their time on social media which can affect their communication with people in real life or affect their sleep patterns which can have a detrimental effect on their mental health. Young people often have completely unrealistic views on everything from career choices to their own personality (hair, clothes, etc.)... Many young people only communicate through socials and often lose confidence and soft skills when talking 'in real life'. Also many young people have misconceptions on attitudes about various things as often opinions or differences of opinions can often be misunderstood when just in a txt or meme etc." |  | Discusses social media's influence on real-life communication skills and confidence in young people, emphasising social work's role in addressing challenges and promoting healthy social development. |
|  | **Self-Esteem and Body Image**: Many headteachers note the negative impact of social media on self-esteem and body image, particularly due to exposure to unrealistic standards and constant comparison. | "Social media use among these age groups has an intense influence on their mental health. In general, kids, particularly younger ones, have a deep need to belong socially and being able to conform to group ideals - or not being able to - can profoundly affect a social media user’s self-image which in turn is inextricably linked with mental health. Social media can intensify the formation of both in-groups and out-groups and children and adolescents who find themselves in the latter in particular are especially vulnerable to negative mental health effects from cyberbullying and lack of self-esteem" |  | Underscores the need for belonging and conformity among young people, crucial aspects for social workers in supporting healthy self-concept development and addressing mental health issues linked to self-image. It also highlights the vulnerability to cyberbullying and the role of social media in shaping in-groups and out-groups, valuable insights for social work practice. |
|  |  | “Social media has a massive influence on them. Children and young people are far more conscious of their appearance now and as a result are wearing makeup much younger (females) or are spending time in the gym building muscles (males) compared to 20 years ago. They also have to have access to their phones at all times and can't leave a snap unanswered or unopened for fear of missing out"​ |  | Underscores social workers' role in helping young people develop self-worth independent of social media metrics, addressing the stress of constant online presence, body image issues, and Fear-Of-Missing-Out-related pressures. |
|  | **Cyberbullying and Online Harassment**: Cyberbullying emerges as a significant concern, with social media providing a platform for persistent and sometimes anonymous bullying | “Online bullying - students can be reached 24/7 by those meaning to cause them harm. Peer pressure/ rumours” |  | Emphasises the continuous nature of cyberbullying via social media, underscoring the need for interventions addressing digital resilience and support for affected children. |
|  |  | The bullying that occurs on social media permeates all aspects of a child's life so that they can't switch off from it and so it can destroy mental health. |  | Emphasises the all-encompassing effects of cyberbullying on a child's life and the destructive impact on mental health, underscoring the urgency for social workers to provide comprehensive support and intervention. |
|  | **Information and Misinformation**: The role of social media as a primary source of information for young people, often without a balanced perspective, is highlighted. | "The kind of language used in interactions is awful sometimes and the misinformation they get from [*named social media*] in particular is dreadful." |  | Discusses the negative impact of misinformation on young people's language and interactions through social media, highlighting the importance of social work interventions to promote media literacy and critical thinking skills. |
|  |  | "These are impressionable minds yet typically only hear one side of an argument. Young people can also be 'empowered' by the fact it's so easy to post; bigging the poster up and potentially causing unknown mental harm to the 'victim' in the post and/or recipient of a post." |  | Emphasises the potential for harm in one-sided online arguments and the importance of guiding young people to understand their online actions and develop empathy, a crucial aspect of social work in promoting healthy digital habits and addressing digital communication's psychological and social implications. |
|  | **Addictive Behaviours and Mental Health**: The addictive nature of social media and its impact on various aspects of mental health, such as anxiety, depression, and stress, is frequently mentioned. | "The impact of social media on mental health is complex and multifaceted with both positive and negative effects. Negative: Addiction and problematic use: Excessive social media use can become addictive, interfering with daily life, sleep, and academic or professional responsibilities. Problematic social media use can also lead to social isolation and withdrawal from other meaningful activities"​ |  | Highlights the addictive nature of social media, its interference with daily life, the link to social isolation, and its complex impact on mental health. Social workers need to address these issues effectively. |
|  |  | "It has a negative impact on mental health. It doesn't allow the young person to form meaningful connections and there is no definition between virtual and reality. Addiction is another- the pursuit of likes and engagement providing a temporary high of sorts plus fear of missing out and not responding to/viewing notifications. Social media interactions impact organic friendship development and facilitate superficial relationships that leave young people feeling connected but lonely"​ |  | Underscores the impairment of meaningful social connections, the blurring of virtual and real life, 'likes' addiction, FOMO, and the impact on friendship and loneliness—crucial areas for social work intervention and community integration. |
| Anthropology | **Digital Culture and Identity Formation**: This reflects how digital culture impacts self-representation and identity. | "Students have no 'switch off' time and live a great proportion of their lives through social media. A lot of students base their identity off their 'profiles' and 'likes' - which is a shame as they often see it as a measure of their 'worth'" |  | Demonstrating how social media shapes student identity. It signifies a shift in identity formation from traditional to digital realms, highlighting virtual interactions' impact on self-concepts and social status. |
|  |  | "Some use [social media] as an alternative to self-harm or as a means to manage difficult emotions while others seek inspiration from social influencers. Additionally, young individuals employ social media for personal business ventures, showcasing their talents and creating virtual identities for self-exploration" |  | Diverse roles of social media in the lives of young people, from emotional management to self-expression. Anthropologically, it reflects digital culture's integration into identity formation processes. |
|  | **Developmental Impacts of Digital Engagement:** Indicating how persistent digital engagement could influence neurological and psychological development. | “The algorithms in social media are quite problematic for people of all ages. In general, social media contributes to a lack of concentration and a shortening of the attention span in people, possibly leading to ADHD. There is also an unhealthy relationship with one's own public image and how one shows oneself to others, since social media promotes showing off to others and being too open about one's private life. The younger the person is exposed to social media, the stronger the consequences on their mental health.” |  | Insights into the developmental impacts of persistent digital engagement, potentially affecting neurological and psychological development in young people. |
|  |  | "An excessive reliance on social media as a coping mechanism can result in isolation from physical support networks. Some young people may avoid face-to-face communication and struggle to filter out negative content which can adversely affect their mental well-being... social media use has a strong influence on behaviour and mental health in all the indicated ages. It affects the development of their capabilities and I see evident negative effects on their emotional regulation" |  | Underscores the significance of anthropological perspectives by showcasing the transformative effects of deep digital media engagement, particularly in reshaping socialisation and interpersonal dynamics, with implications for individual and societal development. |
|  | **Communication Patterns and Language Use.** Showing the shift in language use and communication norms. | "social media has become a means of interacting and communicating with others that has no time or distance limitations. This has meant bullying can occur at any point in time on any day... we start to blur boundaries of what is real and what is not and understanding the lived experience of many young people now focuses on online connections through social media rather than physical connectedness" |  | Highlights the transformation of communication patterns through social media, indicating a shift in how humans form and maintain connections, a significant aspect in anthropology, particularly within digital culture.. |
|  |  | "Social media use can have both a positive and negative influence on people's mental health. For those living in isolated areas it can be a great way of keeping in touch with friends and family which would have a positive impact. However, it is easy for young people to be exposed to cyberbullying, inappropriate media content, and the pressure of trying to live up to the unrealistic expectations of so-called influencers. All of this can negatively impact their mental health |  | Signifies the change in communication patterns due to social media's omnipresence and the blurring of virtual and real worlds, impacting socialisation and reality perception. |
|  | **Social Media as a Social Structure**: Illustrating how social media forms a distinct social environment with its own rules and impacts. | "It is consuming and for some the most secure or only source of information or companionship. It is deskilling young people and removing communication and the ability to navigate healthy and relations. Equally it is becoming the source of research and the 'gospel according to' where other more balanced sources exist" |  | Underscores social media's role as a dominant social environment, reshaping communication and information access, affecting social structures and perspectives within the digital realm. |
|  |  | "It provides a platform of what young people aspire to be like. However, it can be that they feel they compare themselves and do not feel they live up to the expectations of what they see. This can lead to negative feelings in young people that they are not good enough. This can then lead to anxiety and depression and low mood. Young people do not realise that social media is not a true reflection of life |  | Illuminates how social media establishes aspirational norms, impacting self-perception, mental health, and reality distortion among young users. |
| Health Sciences | **Psychosocial Impact**: This theme relates to the influence of social media on the psychological and social well-being of young individuals. It includes aspects like self-esteem, social skills, and mental health. For example, many headteachers mentioned social media's role in promoting unrealistic standards and its potential for cyberbullying. | "Social media has a huge impact on young people's mental health. They only see the highlight reel of people's lives and a highly edited version of reality eg edited pictures from influencers. this has a negative impact on body image and general mental health. there is also no limit to what they can access they can be subjected to online bullying. suicide rate particularly in young women has increased since [*named social media*]" |  | Underscores the psychosocial impact of social media on body image, self-esteem, and mental health, including the alarming increase in suicide rates among young women associated with [*named social media*] use. |
|  |  | I strongly believe that social media has a detrimental impact on children's mental health as it creates false perceptions of body image that coerces and influences children to follow 'influencers' opinions and social trends. Social media has also let to a huge spike in online bullying as a result of online spats between children. We have also seen a significant increase in the number of indecent images of children being distributed and issues associated with online grooming" |  | Highlights how social media fosters unrealistic beauty and lifestyle standards, leading to negative self-perception and mental health problems in children. Online bullying, indecent image sharing, and grooming pose serious threats to the social skills, safety, and mental well-being of young individuals, potentially causing psychosocial issues such as anxiety, depression, and distorted self-worth. |
|  | **Behavioural and Habitual Influence**: This covers the addictive nature of social media and its impact on daily habits and behaviours, like sleep patterns and physical activity. Several responses highlighted the addictive quality of social media and its interference with other healthy activities. | "It affects their self image and self worth. It is addictive and causes children and young adults to feel that their life is not ‘perfect’ as social media portrays others in this way" |  | Highlights social media addiction's impact on self-perception and self-worth among children and young adults. It emphasises the compulsive behaviour associated with ongoing social media engagement, which has implications for mental health and social development in health sciences. |
|  |  | "It has an effect on sleep as they are on social media late at night therefore do not get chance to switch off and relax. Self esteem is affected when they see images that have been filtered to look 'perfect'" |  | Underscores how social media disrupts sleep patterns, affecting relaxation and overall health. Exposure to 'perfect' images can distort self-image, impacting self-esteem. It highlights the significant health impact of social media on children and young adults. |
|  | **Emotional Well-being and Mental Health**: This theme encompasses the direct effects of social media on mental health, including stress, anxiety, depression, and overall emotional well-being. The comments often cited concerns about increased anxiety and pressure to conform to social media standards. | "It provides a platform of what young people aspire to be like. However it can be that they feel they compare themselves and do not feel they live up to the expectations of what they see. This can lead to negative feelings in young people that they are not good enough. This can then lead to anxiety and depression and low mood. Young people do not realise that social media is not a true reflection of life"​​. |  | Relevant as it addresses social media's impact on young people's mental health. It highlights how it can lead to unrealistic aspirations, inadequacy, anxiety, depression, and low mood. |
|  |  | "Social media use among these age groups has an intense influence on their mental health. In general kids particularly younger ones have a deep need to belong socially and being able to conform to group ideals - or not being able to - can profoundly affect a social media user’s self image which in turn is inextricably linked with mental health. Social media can intensify the formation of both in-groups and out-groups and children and adolescents who find themselves in the latter in particular are especially vulnerable to negative mental health effects from cyberbullying and lack of self esteem” |  | Highlights social media's significant impact on the mental health of children and adolescents, particularly in terms of social belonging and self-image, which are crucial in health sciences discussions on emotional well-being. |
|  | **Information and Perception Management**: This relates to how social media shapes young people's perceptions and understanding of the world, including body image and lifestyle expectations. There were mentions of social media creating unrealistic life and beauty standards. | "Social media has a huge impact on young people's mental health. They only see the highlight reel of people's lives and a highly edited version of reality eg edited pictures from influencers. this has a negative impact on body image and general mental health. there is also no limit to what they can access they can be subjected to online bullying. suicide rate particularly in young women has increased since [*named social media*]" |  | Highlights the influence of social media on body image and lifestyle expectations, emphasising the potential for unrealistic standards and their impact on mental health, particularly among young women. |
|  |  | "…..of peer pressure unrealistic body expectations/dysmorphia bullying and anxiety as a result of social media. Students have no 'switch off' time and live a great proportion of their lives through social media. A lot of students base their identity off their 'profiles' and 'likes' - which is a shame as they often see it as a measure of their 'worth'" |  | Emphasises how social media shapes self-perception, body expectations, and behaviour in young people, leading to body dysmorphia, bullying, and anxiety, with constant engagement preventing disengagement. It highlights social media's significant role in altering perceptions, pertinent to health sciences. |
|  | **Community and Support Systems**: Some responses acknowledged the positive aspect of social media in providing a sense of community, support, and belonging, especially for marginalised or isolated individuals | "Social connection and support: Social media can provide a sense of belonging and community especially for those who may feel isolated or marginalised. Online platforms can facilitate peer-to-peer support particularly for individuals facing similar challenges or mental health conditions" |  | Acknowledges social media's positive role in fostering a sense of community and support, especially for marginalised individuals, aligning with Health Sciences' focus on promoting mental health and resilience. |
|  |  | "On the other hand the internet and social media can and do help them to find a community that supports them and makes them feel less lonely. They can relate to others find new peers with the same interests and support each other. What's more on the internet they can learn many important things that are overlooked by the school or parents such as mental health education" |  | Highlights the positive role of social media in connecting young people with supportive communities and providing access to important information, aligning with Health Sciences' focus on emotional well-being and mental health. |
