# Supplementary material for: Synthesizing perspectives: Crafting an Interdisciplinary view of social media’s impact on young people’s mental health
Source: PLoS One. 2024 Jul 15;19(7):e0307164. doi: 10.1371/journal.pone.0307164 (PMC11249244; doi:10.1371/journal.pone.0307164)
Supplement: Attachment — Submitted filename: Response to Reviewers.docx [file pone.0307164.s006.docx]

**RESPONSE TO REVIEWERS**

Thank you for considering the recent submission and for your encouraging response regarding the revision and resubmission of the manuscript. I extend my gratitude to the reviewers for their insightful, helpful, patient, and constructive feedback. In response to the reviewers' comments, I have thoroughly reviewed the paper and made numerous significant changes and additions. I have also corrected some typing errors and I apologise for the presence of those. To provide clarity the revised text corresponding to each response is included alongside the response with the location of the change in the revised manuscript also presented.

**General Comments**

1. **Please include your full ethics statement in the ‘Methods’ section of your manuscript file. In your statement, please include the full name of the IRB or ethics committee who approved or waived your study, as well as whether or not you obtained informed written or verbal consent. If consent was waived for your study, please include this information in your statement as well.**

>>>>>This is now provided on Page 7, Lines 1 to 7 of the revised manuscript as follows:

“Ethics approval for the study was granted by the University of Leicester Psychology Ethics Board, with the reference number 42292-jm148-ls&visionsciences,schof. In alignment with ethical guidelines, informed consent was obtained from all participants. This consent was facilitated through an online survey, where respondents indicated their consent by affirmatively selecting the consent option on the digital form confirming consent statements. This consent option confirmed that participants were fully informed and voluntarily agreed to participate in the study. “

2. **Please include a caption for figure 1.**

>>>>>> Apologies there is no Figure 1. This is an error – and is Table 3. Any reference has been removed.

3. **Please upload a copy of Figure 1, to which you refer in your text on page 23. If the figure is no longer to be included as part of the submission please remove all reference to it within the text.**

>>>>>> Apologies there is no Figure 1. This is an error – and is Table 3. Any reference has been removed.

4. **Please review your reference list to ensure that it is complete and correct. If you have cited papers that have been retracted, please include the rationale for doing so in the manuscript text, or remove these references and replace them with relevant current references. Any changes to the reference list should be mentioned in the rebuttal letter that accompanies your revised manuscript. If you need to cite a retracted article, indicate the article’s retracted status in the References list and also include a citation and full reference for the retraction notice.**

>>>>> We have reviewed the reference list, double-checked it for errors, and updated it. Additionally, in response to Reviewer 2's concern (point 3) about the lack of literature, we have revised the manuscript to include over 50 more references.

**Editor comments**

**The reviewers primarily highlighted the need for enhancements in the article's theoretical and methodological aspects. Their observations provide valuable insights to fortify the study's theoretical foundation and methodological robustness.**

**I would like to request that all adjustments and requests highlighted by the reviewers be addressed in the new version of the article. This encompasses, but is not confined to:**

1. **Revising and deepening the theoretical framework, ensuring the consideration and coherent integration of all pertinent approaches.**

>>>>> Our theoretical framework now emphasizes the integration of multiple disciplines to develop a more holistic understanding of social media's impact on mental health, recognizing the complexity of these interactions. The literature review has been significantly expanded to include over 50 new references, providing a robust theoretical foundation. Theoretical insights have been translated into practical, policy-relevant recommendations. We provide specific strategies for integrating digital literacy into curricula, guidelines for mental health interventions, and policy recommendations to address the challenges posed by social media. We have added a detailed discussion of the strengths and limitations of the Comprehensive Digital Influence Model. This section highlights the robustness of the model in capturing the complex dynamics between social media and mental health among young people.

By revising and deepening the theoretical framework through these comprehensive and interdisciplinary approaches, we ensure a more nuanced and holistic understanding of social media's impact on young people's mental health, thereby enhancing the persuasiveness of the article.

2. **Enhancing the methodology, which entails clarifying procedures, justifying methodological choices, and adequately addressing potential limitations.**

>>>>> The methodology has been clarified and enhanced by providing a more in-depth justification for using ChatGPT-4, including references to relevant studies that support its application in qualitative research. We addressed concerns about its unbiased processing capability by discussing its limitations and citing studies that highlight potential biases. This includes a discussion of political biases in ChatGPT-4 and their minimal expected influence in this context.

3. **Integrating any additional suggestions or comments provided by the reviewers to enhance the overall quality of the article.**

As outlined below we have fully considered these comments and are detailed below, but summarised here as follows:

**Reviewer #1:**

- **Key Themes Robustness:** We have added a discussion on Page 33, Lines 1-12 about the robustness of the Comprehensive Digital Influence Model and potential areas for refinement, including the integration of developmental psychology, direct input from children and adolescents, and accounting for socio-economic and cultural variables.
- **Practical Implications:** The practical implications section has been expanded on Page 30, Line 15 to Page 32, Line 2, providing specific, actionable recommendations for educators, policymakers, and mental health professionals.

**Reviewer #2:**

- **Research Question:** A research question has been added at the end of the Introduction on Page 6, Lines 16-24 to clearly define the scope of our research.
- **Methodological References:** We provided references and a more in-depth discussion justifying the use of ChatGPT-4, addressing its potential biases, on Page 9, Line 22 to Page 10, Line 25.
- **Results and Discussion:** We expanded the Results and Discussion sections to include over 50 new references, developing a critical analysis of the literature on Pages 12-13 and Pages 26-27, and comparing primary and secondary school analyses on Pages 29-30.
- **Interdisciplinary Approach:** The value of the interdisciplinary approach is highlighted in the Introduction and Discussion sections on Pages 5-6 and Pages 30-32, demonstrating how it provides a more holistic and nuanced understanding compared to single-discipline studies.

4. **I underscore the importance of these revisions to ensure that the article fulfills its maximum potential and merits consideration for publication in our journal.**

>>>>> We have meticulously addressed each reviewer's suggestions, significantly enhancing the theoretical framework, methodology, and integration of interdisciplinary perspectives. We think these revisions ensure that the article presents a comprehensive, nuanced analysis of the complex interplay between social media and young people's mental health, making it a valuable resource for stakeholders and worthy of consideration for publication.

**Reviewer #1:**

1. **Regarding the Comprehensive Digital Influence Model, how robust are the identified key themes in capturing the complex dynamics between social media and mental health among young people? Are there any potential limitations or areas where further refinement may be necessary?**

>>>> We acknowledge the need to further elaborate on the robustness of the identified key themes in capturing the complex dynamics between social media and mental health among young people. To address this, we have added a section discussing the strengths and limitations of the Comprehensive Digital Influence Model, highlighting areas for potential refinement and future research. The revised section is on Page 33, Lines 1 to 12, as follows:

“As such there are three possible ways in which this can be done to refine and develop the model. First, integrating developmental psychology more explicitly could provide a deeper alignment of the model with the cognitive, emotional, and social development stages of children and adolescents. This approach would benefit from the inclusion of insights from developmental psychologists to refine how social media impacts are framed within different age groups. Second, expanding data sources to include direct input from children and adolescents through qualitative studies or surveys could enrich the model by incorporating firsthand perspectives on social media's influence, adding depth and accuracy. Lastly, refining the model parameters to account for variables such as socio-economic status, cultural background, and individual psychological traits would enable the creation of more nuanced interventions and policies, tailored to meet the diverse needs of different student populations, thereby enhancing the model’s utility and applicability.”

2. **In terms of practical implications, how do the insights provided by the study inform the actions of educators, policymakers, and mental health professionals? Are there specific recommendations or strategies proposed that are actionable and feasible in addressing the challenges posed by social media in supporting the mental well-being of young individuals?**

>>>>> The discussion on practical implications has been expanded to provide specific, actionable recommendations for educators, policymakers, and mental health professionals. We outline strategies for integrating digital literacy into curricula, guidelines for mental health interventions, and policy recommendations to address the challenges posed by social media. The revised section is on Page 30, Line 15 to Page 32, Line 2, as follows:

“The study delivers significant insights across various domains, notably by establishing a more holistic, nuanced understanding of the complex interplay between social media and young people's mental health, and by formulating succinct, policy and educationally relevant recommendations. Employing ChatGPT-4 for thematic analysis, our methodology introduces a comprehensive multidisciplinary lens to examine the complex interactions between social media use and the mental health of young individuals. This innovative approach not only enhances the granularity of our findings but also establishes a new precedent for future research methodologies within this sphere. The introduction of the Comprehensive Digital Influence Model—based on the study’s findings—provides a structured, holistic framework that encapsulates the multifaceted impacts of social media across different developmental stages and thematic areas. By synthesizing perspectives from diverse disciplines such as sociology, psychology, education, and anthropology, the model offers a more holistic and complex understanding of social media's influence than is typically achieved through single-discipline studies. This comprehensive approach is especially valuable for mental health professionals and educators, equipping them with a nuanced understanding that can inform therapeutic interventions and educational curricula designed to mitigate the risks associated with social media use, such as cyberbullying and addiction. Regarding policy implications, our findings are instrumental for policymakers in shaping educational content that addresses critical areas like digital literacy, mental health, and social skills, tailored to cater to various developmental needs. The study highlights the importance of creating informed governance and policies that effectively manage and regulate digital media's influence on youth, advocating for partnerships with technology companies to establish safer online environments. It also suggests enhancing parental and community involvement to better manage children’s digital interactions, emphasizing a collaborative approach to digital education and safety. Furthermore, the insights derived from this research are crucial for the development of educational practices that foster not only digital literacy but also critical thinking and social-emotional learning. By integrating these elements into school curricula, the study advocates for a more informed and proactive approach to social media use among young people. This includes empowering students to critically assess online content and engage in positive online interactions, thereby equipping them with the tools needed to navigate the complexities of digital spaces effectively. In essence, this study offers valuable contributions to theoretical frameworks, policy development, and educational practices, underscoring the need for an integrated approach that combines insights from multiple disciplines to fully address the intricate dynamics of social media’s impact on young people’s mental health. This makes it a pivotal resource for stakeholders ranging from academic researchers to policymakers and educators, all of whom play a critical role in shaping the digital landscape to support the well-being of young users.”

**Reviewer #2:**

1. **Include a Research Question (RQ): The first chapter (Introduction) is effective in showing the topic and clearly stating the research gap, highlighting the lack of studies exploring the effect of social media on young people's mental health from an interdisciplinary perspective. However, it would be advisable to include a research question at the end of this chapter, aiming to state the research scope.**

>>>>> A research question has been added at the end of the Introduction to clearly define the scope of our research. The revised section is on Page 6, Lines 16 to 24, as follows:

“This study aims to delve into the effects of social media on young people's mental health by merging insights from various academic fields. By synthesizing knowledge from disciplines such as psychology, sociology, education, and media studies, we form a holistic understanding of how social media influences the well-being of young individuals. Specifically, this study seeks to answer the research question: 'How do interdisciplinary perspectives enhance our understanding of the impact of social media on the mental health of young people?' This question aims to clarify the scope of our research and guide the investigation into the complex dynamics of social media's influence across different disciplinary lenses.”

2. **Methods: Page 8 contains the following excerpt: “(...) ChatGPT offers a consistent, unbiased processing capability, crucial for handling the subtle and often subjective nature of qualitative data (...).” Considering the use of ChatGPT is the core of the paper’s research design, there is a lack of references/citations that support the author's choice in the use of this tool. In that excerpt, it was mentioned the “unbiased processing capability” of ChatGPT; certainly, that is a questionable statement that demands a more in-depth discussion to justify the use of ChatGPT.**

**Here are some examples of studies that doubt the “unbiased processing capability” of ChatGPT:**

[1] **Rozado, D. (2023). The political biases of chatgpt. Social Sciences, 12(3), 148.**
[2] **Ray, P. P. (2023). ChatGPT: A comprehensive review on background, applications, key challenges, bias, ethics, limitations and future scope. Internet of Things and Cyber-Physical Systems.**
[3] **Motoki, F., Pinho Neto, V., & Rodrigues, V. (2024). More human than human: Measuring ChatGPT political bias. Public Choice, 198(1), 3-23.**

>>>>> Thank you for the details and references. We have provided a more in-depth justification for using ChatGPT-4, including references to relevant studies that support its application in qualitative research. Additionally, we address concerns about its unbiased processing capability by discussing its limitations and citing studies that highlight potential biases (Rozado, 2023; Ray, 2023; Motoki et al., 2024). The revised section is on Page 9, Line 22 to Page 10, Line 25, as follows:

“We propose a qualitative multidisciplinary analysis method for analyzing headteachers' perspectives on social media's influence on young people’s mental health. This method employs thematic analysis across multiple disciplines, followed by the creation of superordinate themes. By integrating thematic analysis across various disciplines and subsequently synthesizing superordinate themes, this approach not only harnesses the power of interdisciplinary insights but also leverages the capabilities of ChatGPT-4 to elevate the process to new levels of precision and depth. ChatGPT-4 excels in a wide array of natural language processing tasks, demonstrating significant capabilities in text summarization, and performs well on natural and large language inference tasks, showing comparable performance to other state-of-the-art models in question-answering and summarization tasks (39–41). This methodology stands out for its comparison to traditional qualitative analysis techniques, primarily through its employment of ChatGPT-4 as a tool for data interpretation. Comparable to content and assessment tasks completed by humans (42,43), ChatGPT-4 potentially offers a consistent, unbiased processing capability, crucial for handling the subtle and often subjective nature of qualitative data. Consequently, when compared to a set of human researchers, ChatGPT-4's advanced algorithms enable it to dissect and interpret quotes within a multitude of academic contexts, ensuring that every piece of data is analyzed thoroughly and from multiple perspectives. It can also handle tasks or recognize objects, concepts, or activities that it has not seen during training. This zero-shot learning capability is particularly valuable because it enables the model to generalize from its existing knowledge without needing explicit examples of every possible scenario it might encounter. However, despite improvements (44), it still retains some level of political bias influenced by factors like the languages used in the system (45–47). This could affect its neutrality in applications, presenting a caveat in the deployment of this model in multidisciplinary qualitative analysis where unbiased perspectives are critical. It was felt that the potential political bias of ChatGPT-4 is less concerning when analyzing headteachers' views on social media's impact on youth mental health. The focus on educational outcomes, reliance on professional insights, and broad agreement on the importance of addressing youth mental health issues help minimize the influence of any bias.”

>>>>> We have also acknowledged this as part of the limitations in the discussion of findings. The revised section in on Page 32, Lines 12 to 18 as follows:

“Methodologically, while the use of ChatGPT-4 for thematic analysis is innovative, it might have intrinsic limitations in fully grasping the qualitative nuances that human researchers, particularly those with a deep understanding of educational contexts, could interpret. Moreover, it's important to consider the political biases noted in ChatGPT-4 (45–47), as these may influence how it processes and interprets data. While the influence of such biases is expected to be limited in the context of analyzing educational impacts of social media on mental health among young people, they are still worth careful consideration.”

3. **Results are essentially descriptive, and there is a lack of discussion of findings with the literature: The main weakness of the paper relies on the descriptive aspect of the chapters Results and Discussion. In essence, these chapters describe the outputs from the thematic analysis without any analysis or discussion with the previous literature on the impact of social media on young people's mental health (from a disciplinary or interdisciplinary perspective) (there is no citation from pages 12 to 26).**

>>>>> We have developed both of these areas and the paper generally by revising it to be more critically considered and introducing over 50 new references to support the points. In response to the reviewers' comments, there are two key areas in which we develop the critical aspects within the literature. The first area outlines the literature that defines the 10 disciplinary areas used in the study. This is provided on Page 12, Line 20 to Page 13, Line 14, as follows:

“The reason for including these 10 areas was as follows. **Psychology**: Crucial for understanding the cognitive, emotional, and behavioural impacts of social media on the mental health of children and adolescents (48–50). **Sociology**: Provides insights into how social media shapes and is influenced by social structures, norms, and interactions, impacting the mental health of young individuals (13,51). **Education Studies**: Offers perspectives on the role of social media in educational environments and its influence on learning, development, and mental health of students (52). **Political Science**: Examines the impact of social media on young people’s political socialisation, civic engagement, and its broader implications for their mental well-being (53,54). **Philosophy**: Explores ethical, existential, and epistemological aspects of social media use, contributing to a deeper understanding of its impact on young people’s mental health (55,56). **Media Studies**: Investigates the role of media content, technology, and communication patterns in shaping young people’s perceptions, behaviours, and mental health (57). **Linguistics**: Analyses how language use and communication in social media influence the mental health, identity, and social interactions of young people (58). **Social Work**: Addresses the practical implications of social media use on young people’s mental health and provides frameworks for intervention and support (59,60). **Anthropology**: Offers insights into the cultural and societal dimensions of social media use and its impact on the mental health of children and young adults (61). **Health Sciences**: Focuses on the physiological and psychological aspects of social media use, examining its effects on the overall mental and physical health of young people (62,63).”

>>>>> A second area relates to describing the common themes of the introduced model within the relevant literature. This is provided on Page 26, Line 1 to Page 27, Line 2 as follows:

“The analysis of responses from both primary and secondary school headteachers revealed common themes, leading to the development of a new model, we name as the Comprehensive Digital Influence Model (See Table 3), focusing on five key areas:

- Self-Identity and Perception Formation: Both primary and secondary levels emphasise the significant role of social media in shaping self-image, identity, and self-esteem. This convergence indicates a universal effect of social media in moulding young people’s self-conception across all ages (66–70).
- Social Interaction Skills and Peer Communication: A shared concern is the impact of social media on social skills and peer interactions, including the prevalence of cyberbullying. This highlights a widespread apprehension about the transformation of traditional social dynamics due to digital interactions, evident in both educational stages (71–73).
- Mental and Emotional Well-Being: The analyses from both primary and secondary levels acknowledge the complex relationship between social media use and mental health issues like anxiety and stress. This mutual focus underscores the recognition of social media as a pivotal factor in young people’s mental health, with both its positive and negative aspects (20,74).
- Digital Literacy, Critical Thinking, and Information Perception: There is an emphasis on the necessity for enhanced digital literacy and critical thinking skills. This reflects a common understanding of the importance of empowering young people with the skills to navigate and critically evaluate digital content (75–78).
- Governance, Policy, and Cultural Influence in Digital Spaces: Both educational levels underscore the importance of governance and policy in managing digital spaces and their influence on mental health. This convergence suggests a consensus on the need for structured regulation and guidance in the digital realm, highlighting the crucial role of governance and societal influence in mitigating the challenges posed by digital media (79–81).”

>>>>> The third area describes the comparison of the primary and secondary school analysis, exploring these within the current literature. This is provided on Page 29, Line 1 to Page 30, Line 14, as follows:

“Within the Comprehensive Digital Influence Model (also see Table 3), we are also able to note that in terms of age-related psychological development, the thematic focus of primary and secondary schools diverges notably. Primary school themes are predominantly centred on foundational aspects, encompassing the formation of basic social skills, children's initial interactions with digital media, and the early impact of social media on self-perception and identity formation. In contrast, secondary school themes explore more intricate psychological and social dynamics. For example, in terms of the first dimension, Self-Identity and Perception Formation, in primary schools the focus is on the basic development of social skills and the initial stages of identity formation. Concerns are predominantly centred on how social media influences the early shaping of self-image and identity in children (82). In secondary schools, a more nuanced view is presented, delving into the interactions between psychological factors, social dynamics, digital literacy, and societal structures. This approach reflects a deeper understanding of older children who are navigating more complex stages of psychological development (83). Second is Social Interaction Skills and Peer Communication. In primary schools, the concentration is on developing basic social skills and mitigating the immediate effects of digital communication, with a significant focus on the immediate impact of cyberbullying (84,85). However, in secondary schools, the focus is more on complex social dynamics. This encompasses the development of skills needed for real-world interactions, understanding the role of digital platforms in social interaction, and grappling with the impact of constant connectivity on mental health (32). Third is: Mental and Emotional Well-Being. In primary schools, the emphasis lies on the direct relationship between social media and mental health. This includes addressing issues like constant connectivity, mental overload, and children's initial exposure to potentially inappropriate content (86). However, in secondary schools, Headteachers offer a more layered perspective of mental health, acknowledging both the positive and negative aspects of social media use. This stance indicates a more profound comprehension of the diverse impacts on adolescent mental health (34). Fourth is Digital Literacy, Critical Thinking, and Information Perception. In primary schools, the focus is on rudimentary stages of developing these skills. Efforts are made to help children discern the difference between reality and digital portrayals, thereby laying the groundwork for media literacy (87). Among secondary schools, the emphasis is on the advanced development of digital literacy. This includes integrating these concepts into the curriculum, and fostering sophisticated information perception and management skills, indicating a deeper engagement with digital content (88,89). Fifth is Governance, Policy, and Cultural Influence in Digital Spaces. Among primary schools, a foundational approach is suggested, focusing on the impact of policy, global cultural influences, and the need for enhanced positive parental and community involvement (90). Among secondary schools, headteacher discussions revolve around more sophisticated themes, such as digital governance, the development of policies for digital well-being, and the role of educational leadership in navigating the challenges of the digital era (91,92).”

4. **Explain how the interdisciplinary approach adopted in the paper differs from other studies: The authors mention that the differential of the interdisciplinary approach carried out in the paper is that it allows a more precise analysis of this complex phenomenon, overcoming the limitations of a specialized analysis considering only one field of study. However, the paper**
(i) **lacks a more detailed discussion to demonstrate what would be the differential of the findings using this interdisciplinary approach?**

>>>>> We have addressed this first point in two places. First, in the introduction, we highlight the value of interdisciplinary collaboration, emphasizing a holistic approach, considering complexity, and suggesting nuanced policy and educational recommendations. This is provided on Page 5, Line 5 to Page 6, Line 15, as follows:

“The complex relationship between social media use and young persons' mental health necessitates a multidisciplinary approach to fully comprehend and effectively address its multifaceted nature. The lack of integration among various academic disciplines often results in fragmented insights, with disciplines such as sociology, psychology, education, media studies, health sciences, political science, social work, anthropology, philosophy, and linguistics operating in silos, leading to a partial and segmented understanding. This division hinders the development of comprehensive interventions and policy strategies. To overcome these limitations, an integrated, interdisciplinary perspective is crucial – that would aim for a more holistic account of the topic, recognising the complexity, and present a more succinct account appropriate for policy and educational intervention. First, combining different disciplines could provide a more holistic understanding of impact. For example, whereas a psychology-focused study might exclusively examine the direct effects of social media on individual mental health symptoms like anxiety and depression (29), an interdisciplinary approach by integrating psychology, sociology, media studies, and education offers a broader understanding that encompasses not only these direct effects but also the underlying social dynamics, educational impacts, and media interactions (30–32). This holistic view reveals how these areas intersect and influence one another, leading to more nuanced findings. Second, using different disciplines might acknowledge where levels of complexity exist. For example, while an anthropology-focused study might limit itself to exploring cultural impacts and identity formation (24), combining anthropology with psychology and sociology allows for an exploration of how cultural impacts influence mental health and social behavior in a feedback loop (33). Such an interdisciplinary approach acknowledges complex relationships, for instance, how cultural norms around social media usage can exacerbate or mitigate mental health issues. Third, work then that aims to support educational/policy recommendations also benefit from this interdisciplinary synergy. Instead of a narrow focus from political science on governance and regulation (34), merging insights from political science, social work, health sciences, and media studies allows for the formulation of more nuanced policy recommendations (35). These can address not just the regulation of digital content but also the use of social media to promote mental health resources, community engagement strategies, and educational reforms (8), thereby enriching societal and cultural implications. In summary, the interdisciplinary confluence is imperative not only for addressing the symptoms of social media's influence but also for achieving a cohesive and deeper understanding of how social media shapes both individual identities and broader societal dynamics. This will be accomplished by developing a holistic view, recognizing the complexity of these interactions, and providing succinct, actionable policy and educational recommendations.”

*>>>>>* We have also continued this consideration and thematic structure in the discussion, emphasizing the need to establish a holistic and policy-driven understanding that accounts for the complexity of the area. This is provided on Page 30, Line 15 to Page 32, Line 2, as follows:

“The study delivers significant insights across various domains, notably by establishing a more holistic, nuanced understanding of the complex interplay between social media and young people's mental health, and by formulating succinct, policy and educationally relevant recommendations. Employing ChatGPT-4 for thematic analysis, our methodology introduces a comprehensive multidisciplinary lens to examine the complex interactions between social media use and the mental health of young individuals. This innovative approach not only enhances the granularity of our findings but also establishes a new precedent for future research methodologies within this sphere. The introduction of the Comprehensive Digital Influence Model—based on the study’s findings—provides a structured, holistic framework that encapsulates the multifaceted impacts of social media across different developmental stages and thematic areas. By synthesizing perspectives from diverse disciplines such as sociology, psychology, education, and anthropology, the model offers a more holistic and complex understanding of social media's influence than is typically achieved through single-discipline studies. This comprehensive approach is especially valuable for mental health professionals and educators, equipping them with a nuanced understanding that can inform therapeutic interventions and educational curricula designed to mitigate the risks associated with social media use, such as cyberbullying and addiction. Regarding policy implications, our findings are instrumental for policymakers in shaping educational content that addresses critical areas like digital literacy, mental health, and social skills, tailored to cater to various developmental needs. The study highlights the importance of creating informed governance and policies that effectively manage and regulate digital media's influence on youth, advocating for partnerships with technology companies to establish safer online environments. It also suggests enhancing parental and community involvement to better manage children’s digital interactions, emphasizing a collaborative approach to digital education and safety. Furthermore, the insights derived from this research are crucial for the development of educational practices that foster not only digital literacy but also critical thinking and social-emotional learning. By integrating these elements into school curricula, the study advocates for a more informed and proactive approach to social media use among young people. This includes empowering students to critically assess online content and engage in positive online interactions, thereby equipping them with the tools needed to navigate the complexities of digital spaces effectively. In essence, this study offers valuable contributions to theoretical frameworks, policy development, and educational practices, underscoring the need for an integrated approach that combines insights from multiple disciplines to fully address the intricate dynamics of social media’s impact on young people’s mental health. This makes it a pivotal resource for stakeholders ranging from academic researchers to policymakers and educators, all of whom play a critical role in shaping the digital landscape to support the well-being of young users.”

(ii) **How are the results presented similar to (or different from) other studies analyzing the impact of social media on young people's mental health?**

*>>>>>>* We elaborate on this in two ways. First, in the introduction, we provide an example of how combining disciplines offers a comprehensive understanding of social media's impact, revealing nuanced interactions between psychology, social dynamics, and education. This is provided on Page 5, Lines 14-21, as follows:

“First, combining different disciplines could provide a more holistic understanding of impact. For example, whereas a psychology-focused study might exclusively examine the direct effects of social media on individual mental health symptoms like anxiety and depression (29), an interdisciplinary approach by integrating psychology, sociology, media studies, and education offers a broader understanding that encompasses not only these direct effects but also the underlying social dynamics, educational impacts, and media interactions (30–32). This holistic view reveals how these areas intersect and influence one another, leading to more nuanced findings.”

>>>>>> Also, in the aforementioned paragraph, this part of the discussion explicitly addresses this issue. This is provided on Page 30, Line 15 to Page 31, Line 6, as follows:

“The study delivers significant insights across various domains, notably by establishing a more holistic, nuanced understanding of the complex interplay between social media and young people's mental health, and by formulating succinct, policy and educationally relevant recommendations. Employing ChatGPT-4 for thematic analysis, our methodology introduces a comprehensive multidisciplinary lens to examine the complex interactions between social media use and the mental health of young individuals. This innovative approach not only enhances the granularity of our findings but also establishes a new precedent for future research methodologies within this sphere. The introduction of the Comprehensive Digital Influence Model—based on the study’s findings—provides a structured, holistic framework that encapsulates the multifaceted impacts of social media across different developmental stages and thematic areas. By synthesizing perspectives from diverse disciplines such as sociology, psychology, education, and anthropology, the model offers a more holistic and complex understanding of social media's influence than is typically achieved through single-discipline studies. This comprehensive approach is especially valuable for mental health professionals and educators, equipping them with a nuanced understanding that can inform therapeutic interventions and educational curricula designed to mitigate the risks associated with social media use, such as cyberbullying and addiction.”
